# Predicting Win‐Loss Probabilities for Composite Time‐to‐Event Outcomes Under The Proportional Win‐Fractions Regression Model

**DOI:** 10.1002/sim.70569

**Published:** 2026-04-28

**Authors:** Lu Mao

**Affiliations:** ^1^ Department of Biostatistics and Medical Informatics, School of Medicine and Public Health University of Wisconsin‐Madison Madison Wisconsin USA

**Keywords:** hierarchical composite endpoints, model checking, net benefit, robust variance estimation, win ratio

## Abstract

For composite time‐to‐event outcomes, the win ratio as a relative measure ignores ties resulting from non‐occurrence of events, which can obscure important context in regression settings where event rates—and hence the proportion of ties—vary over time and across covariate values. To gain a more complete understanding of covariate effects, we propose coupling the proportional win‐fractions (PW) model, which specifies only the win ratio, with a time‐to‐first‐event model (e.g., Cox model), from which the tie probability can be inferred. This combination enables prediction of time‐dependent win and loss probabilities on an absolute scale for any given pair of covariate profiles, with uncertainty quantified through robust variance estimation, and facilitates inference on tie‐adjusted measures such as the net benefit and win odds to complement the win ratio in evaluating effect size. Residual‐based diagnostics further allow refinement of model fit through appropriate covariate specification or stratification to address potential violations of proportionality assumptions. Through simulation studies and an application to the landmark HF‐ACTION trial, we demonstrate that the proposed approach provides accurate and clinically interpretable predictions when model assumptions are approximately satisfied. These predictions reveal a pattern of diminishing returns on absolute win–loss probabilities as a key baseline biomarker increases across the population despite a constant win ratio. We also show that violations of the proportional win‐fractions assumption can lead to biased predictions, underscoring the importance of model diagnostics. When the primary objective is to characterize time‐varying covariate effects on win–loss probabilities, more flexible modeling approaches may be warranted. In addition to a small code example in the paper, the full methodology is implemented in the WR package, available on GitHub ( https://lmaowisc.github.io/WR/) and the Comprehensive R Archive Network (CRAN).

## 1 Introduction

The win ratio, introduced by Pocock et al. [1], has emerged as a popular summary measure for composite endpoints in clinical trials [2, 3, 4]. It is particularly appealing when the components are naturally ordered by clinical importance, such as mortality over hospitalization or biomarkers. In comparing outcomes pairwise across groups [5], the win ratio prioritizes important events over lesser ones and provides a simple yet interpretable effect size, expressed as the relative frequency of wins (i.e., more favorable outcomes) to losses between the treatment and control groups.

Building on this framework, our earlier work extended the win ratio for composite time‐to‐event outcomes to incorporate covariates through the proportional win‐fractions (PW) regression model [6]. Specifically, we model a time‐constant win ratio as a multiplicative function of covariate differences, with regression coefficients representing the log‐win ratios due to unit increases in the covariates. The model reduces to the standard two‐sample win ratio when the only covariate is the treatment indicator. In its general form, it allows adjustments of possible confounders and assessments of quantitative risk factors, such as patient age, body mass index, or (baseline) left ventricular ejection fraction, a key predictor in heart failure trials [7].

However, there are intrinsic limitations to the win ratio as a relative measure. The status of win or loss is not a dichotomy. Rather, there is a third category—tie—which happens when neither subject experiences an event during their *shared* observation window [1]. The presence of ties means that the magnitudes of win and loss proportions cannot be inferred from their ratio, which causes important context to be missing. For example, an improvement measured by 20% win vs. 10% loss would be viewed very differently from one measured by 0.2% win vs. 0.1% loss, despite the common fold change. In fact, the latter would likely not be considered clinically meaningful, as it is driven by so few events. This distinction is particularly important in clinical decision‐making, where comparisons are often made between patients with different baseline risk profiles, and absolute probabilities of favorable outcomes over time are needed to guide interpretation.

Motivated by this need to better characterize treatment effects on an absolute scale, alternative metrics such as the net benefit and win odds have been proposed. The net benefit is defined as the difference between the win and loss proportions, offering a contrast in absolute rather than relative terms [5, 8]. The win odds, on the other hand, allocate half of the ties to each of the win and loss proportions, thereby accounting for the impact of ties [9, 10]. These tie‐adjusted measures complement the win ratio by providing a fuller picture of the treatment effect, especially when event rates are low so that ties are more common [11].

While it is straightforward to calculate the win and loss proportions between two groups, it is less clear how to model them coherently in a regression setting. For robustness, the PW model takes a “minimalist” approach to modeling the win ratio, with no separate assumptions on the numerator and denominator. This means that, for a given pair of covariate vectors, their win and loss probabilities cannot be predicted from a fitted PW model alone. In addition, the time‐varying nature of win‐loss probabilities presents unique challenges for building a coherent model, which must account for the interplay between covariate effects and follow‐up time (for the win ratio, this problem is circumvented by the PW model assumption that the win‐loss probabilities, or fractions, are proportional over time) [12].

A naive solution is to model the entire joint distribution of the component events and use it to derive the win‐loss probabilities as functions of time [13]. Not only would this approach be assumption‐heavy, computationally intensive, and hard to scale, but it would also make it impossible to quantify covariate effects in simpler terms. Most importantly, checking model assumptions would be a challenge as they are not targeted towards the quantities of interest (wins and losses). Instead, a more effective approach would involve amending the PW model just enough to enable the extraction of win‐loss probabilities while maximally preserving its parsimony and interpretability.

To take this approach, we can draw on the trinomial nature of win, loss, and tie. Since the PW model already provides the ratio of win vs. loss probabilities, we only need the tie probability to obtain all three of the trinomial parameters. In standard settings, a tie arises when both subjects are event‐free by a certain point. As such, its probability is given by the product of two survival functions for the time to the first event (TFE), which can be predicted using any of the popular (univariate) survival models, such as the Cox proportional hazards (PH) model [14]. Here, although the TFE model is not of direct interest in assessing the possibly hierarchical composite endpoint, it provides complementary information to the PW model to allow mapping of win‐loss probabilities from a relative to an absolute scale.

In this paper, we follow this approach to predict the (time‐dependent) win‐loss probabilities based on the PW model, thereby enabling direct comparisons between subjects with pre‐specified covariate profiles and translating relative effects into clinically interpretable probability summaries over time. In the process, we address technical challenges, including uncertainty quantification for estimators derived from two distinct models and accounting for win‐loss correlations when forming contrasts such as the net benefit and win odds.

Starting with a review of the PW model, Section 2 presents the methodological details of the proposed procedures, focusing particularly on the Cox model as an example of the TFE model. This section also addresses model checking through residual analysis for both the PW and Cox models to ensure reliable predictions. A simple, reproducible code tutorial is provided, demonstrating the newly implemented functionalities in the R package WR. Comprehensive simulation studies, detailed in Section 3, evaluate the performance of the procedures in realistic settings. In Section 4, we analyze data from HF‐ACTION, a landmark cardiovascular trial, to examine the impact of baseline cardiopulmonary exercise (CPX) duration on post‐randomization win‐loss trajectories for the prioritized endpoint of death and hospitalization. Finally, in Section 5, we discuss strategies for building a general regression model for win‐loss probabilities without the constraints of the PW or Cox models.

## 2 Methods

### 2.1 Full Data and Review of PW Models

Following the convention in our previous work, let D denote the survival time and $N_{D}(t)=I(D\leq t)$ the corresponding counting process, where I(·) is the indicator function. For nonfatal events, let $N_{1}(t),\ldots ,N_{K}(t)$ denote the counting processes for K distinct types of (possibly recurrent) events. These nonfatal components may themselves be organized hierarchically, with, e.g., life‐threatening events ranked above less severe symptoms. Overall, let

$$Y(t)=\{N_{D}(u),N_{1}(u),\ldots ,N_{K}(u):0\leq u\leq t\}$$

represent the collection of all event processes up to time t. Additionally, consider a p‐vector of covariates $Z={\left(Z_{\cdot 1},\ldots ,Z_{\cdot p}\right)}^{T}$, which may contain a treatment indicator as well as other risk factors.

Let $Y_{i}(t)$ and $Y_{j}(t)$ denote the outcome processes for two independent subjects i and j, respectively. The PW modeling framework starts with specifying a “win function” $𝒲(Y_{i},Y_{j})(t)=1$ or 0 to indicate whether or not subject i has a more favorable outcome than subject j by time t. As a user‐defined rule of comparison, 𝒲 can be flexible but needs to satisfy the following conditions:

C1. $𝒲(Y_{i},Y_{j})(t)$ is a function of $Y_{i}(t)$ and $Y_{j}(t)$ only;
C2. $𝒲(Y_{i},Y_{j})(t)+𝒲(Y_{j},Y_{i})(t)=0\;\text{or}\;1;$
C3. $𝒲(Y_{i},Y_{j})(t)=𝒲(Y_{i},Y_{j})(D_{i}\wedge D_{j}\wedge t)$ for all t, where x∧y=min(x,y).

These conditions ensure that: (1) the outcomes being compared are evaluated within the same time frame; (2) the result is always classified as a win, loss, or tie; and (3) death, as a competing risk, does not interfere with the determination of the win‐loss result—this holds true if death is prioritized, as it permanently resolves the win‐loss status upon occurrence. For example, in the standard case where death is prioritized over a single nonfatal event, the win function can be expressed as

(1) $$\begin{array}{cc} 𝒲(Y_{i},Y_{j})(t) & =\underset{\text{Win on survival}}{\underbrace{I(D_{j}<D_{i}\wedge t)}}+\underset{\text{Tie on survival, win on nonfatal event}}{\underbrace{I(D_{i}\wedge D_{j}>t,T_{j}<T_{i}\wedge t)}}, \end{array}$$

where T denotes time to the nonfatal event, and the subscript indicates the subject affiliation of the corresponding quantity. It is straightforward to verify that this 𝒲 satisfies conditions (C1)–(C3).

Under this framework, the conditional probability of a win for subject i against subject j by time t, given their covariates $Z_{i}$ and $Z_{j}$, is

(2) $$w(t|Z_{i},Z_{j})=\mathrm{pr}\{𝒲(Y_{i},Y_{j})(t)=1|Z_{i},Z_{j}\}.$$

Likewise, the loss probability for subject i (i.e., win probability for subject j) is $w(Z_{j},Z_{i})(t)$, defined by switching the subscripts in Equation (2). Though time‐dependent, these win‐loss probabilities (or *fractions*) are constrained to be proportional over t under the PW model, resulting in a constant win ratio given by

(3) $$r(t|Z_{i},Z_{j})\equiv \frac{w(t|Z_{i},Z_{j})}{w(t|Z_{j},Z_{j})}=\exp\{\beta^{T}(Z_{i}-Z_{j})\},$$

where β is a vector of coefficients that can be interpreted as the log‐win ratios resulting from unit increases in the covariates. Mao and Wang [6] discussed different scenarios under which (3) holds for common choices of 𝒲.

### 2.2 General Strategy

Our target estimands are $w(t|z,z^{*})$ and $w(t|z^{*},z)$, i.e., the win and loss probabilities comparing (user‐specified) covariate vectors z and $z^{*}$. These vectors most commonly differ in only one component to isolate the effect of a specific covariate, though this is not a strict requirement. Unfortunately, these probabilities cannot be directly estimated from Equation (3) alone, which specifies only their ratio. However, if we posit an additional model for the tie probabilities, defined by

$$\begin{array}{ll} \nu (t|z,z^{*}) & =\mathrm{pr}\{𝒲(Y_{i},Y_{j})(t)=𝒲(Y_{j},Y_{i})(t)\\ & =0|Z_{i}=z,Z_{j}=z^{*}\}, \end{array}$$

then the trinomial relationship $\nu (t|z,z^{*})+w(t|z,z^{*})+w(t|z^{*},z)=1$ implies that

(4) $$\begin{array}{ll} w(t|z,z^{*}) & =\{1-\nu (t|z,z^{*})\}r(t|z,z^{*}){\left\{1+r(t|z,z^{*})\right\}}^{-1}\\ & =\{1-\nu (t|z,z^{*})\}\frac{\exp\{\beta^{T}z\}}{\exp\{\beta^{T}z\}+\exp\{\beta^{T}z^{*}\}} \end{array}$$

and that $w(t|z^{*},z)$ is expressed by (4) with z and $z^{*}$ switching places.

Modeling $\nu (t|z,z^{*})$ is simplified by the fact that it is an independent parameter, unconstrained by the magnitude of $r(t|z,z^{*})$. Moreover, in the common scenario where win‐loss is based on the order of continuously monitored events, a tie by time t simply means that both patients are event‐free. Consequently,

(5) $$\nu (t|z,z^{*})=S(t|z)S(t|z^{*}),$$

where $S(t|Z)=\mathrm{pr}(\tilde{T}>t|Z)$ is the conditional survival function for time to the first event (TFE) $\tilde{T}$. We can then fit S(t|Z) using any of the standard regression models (e.g., Cox PH, proportional odds, or AFT models) for $\tilde{T}$ against Z, use (5) to compute $\nu (t|z,z^{*})$, and, finally, obtain the win‐loss probabilities through (4).

Remark 1 The definition of TFE depends on what events are used in 𝒲. If all events in Y(t) are involved in determining wins and losses, then $\tilde{T}=\inf\left\{t:N_{D}(t)+\sum_{k=1}^{K}N_{k}(t)\geq 1\right\}$, i.e., the first time any of the component counting processes jumps to 1. In the case of (1), however, $\tilde{T}=D\wedge T$. Therefore, the TFE should be understood as a shorthand for “time to the first event that is used to break ties”.

### 2.3 Estimation and Uncertainty Quantification

In practice, the outcomes are subject to censoring. Let C denote the censoring time that is independent of Y(t) given Z. A random n‐sample of observed data then consists of

(6) $${𝒪}_{i}\equiv \{Y_{i}(X_{i}),X_{i},Z_{i}\},\;i=1,\ldots ,n,$$

where X=D∧C. Suppose we posit a (semiparametric) model S(t|z;η) for $\tilde{T}$, with η denoting the set of parameters, and estimate η by some $\hat{\eta }$ using relevant parts of the observed data (6). Then, by (4) and (5), we can estimate the win‐loss probabilities for any given covariate vectors z and $z^{*}$ by

(7) $$\hat{w}(t|z,z^{*})=\{1-S(t|z;\hat{\eta })S(t|z^{*};\hat{\eta })\}\frac{\exp\{{\hat{\beta }}^{T}z\}}{\exp\{{\hat{\beta }}^{T}z\}+\exp\{{\hat{\beta }}^{T}z^{*}\}},$$

where $\hat{\beta }$ is an estimator of β under the PW model [6].

Standard software packages are available to implement (7). For Cox, PO, and AFT models for the TFE, e.g., we can use R‐functions survival::coxph(), timereg::prop.odds(), and survival::survreg(), respectively, for model‐fitting and calculation of $\hat{\eta }$. Combined with $\hat{\beta }$ obtained from a PW model fitted using WR::pwreg(), this provides all the quantities needed to calculate $\hat{w}(t|z,z^{*})$.

Estimating the standard error is less straightforward, as it must allow for correlations between two sets of parameters estimated from two distinct models. One way to address this complication is to derive the influence function of $\hat{w}(t|z,z^{*})$ via the delta method on $\hat{\eta }$ and $\hat{\beta }$. In particular, the following quantities will be needed in the asymptotic linear expansion of $\hat{\beta }$:

$$\begin{array}{ll} \text{(Observed win):} & \;\delta_{ij}(t)=𝒲(Y_{i},Y_{j})(t\wedge X_{i}\wedge X_{j}),\\ \text{(Observed comparability):} & \;R_{ij}(t)=\delta_{ij}(t)+\delta_{ji}(t),\\ \text{(Win probability given comparability):} & \;\mu (Z_{i},Z_{j};\beta )\\ & \;=\frac{\exp\{\beta^{T}Z_{i}\}}{\exp\{\beta^{T}Z_{i}\}+\exp\{\beta^{T}Z_{j}\}},\\ \text{(Pairwise win residual):} & \;M_{ij}(t|Z_{i},Z_{j};\beta )\\ & \;=\underset{\text{Observed}}{\underbrace{\delta_{ij}(t)}}-\underset{\text{PW model‐based}}{\underbrace{R_{ij}(t)\mu (Z_{i},Z_{j};\beta )}}, \end{array}$$

Here, “comparability” in this context means that at least one subject in the pair experiences an event by time t, and the pairwise win residual ranges in [−1,1].

We first work out a general expression for the asymptotic linear expansion of $\hat{w}(t|z,z^{*})$, and then tailor the expression to a Cox model for the TFE. Detailed derivations are given in the Supporting Information online.

Proposition 1 *Suppose for a general TFE model*, $n^{1/2}(\hat{\eta }-\eta )=n^{-1/2}\sum_{i=1}^{n}\psi_{\eta }({𝒪}_{i})+o_{p}(1)$ *for some* (*mean‐zero*) *influence function* $\psi_{\eta }$ *as* n→∞. *Furthermore, let* $\hat{\beta }$ *be the estimator that solves the standard PW‐estimating equation* $\sum_{i=1}^{n-1}\sum_{j=i+1}^{n}(Z_{i}-Z_{j})M_{ij}(\infty |Z_{i},Z_{j};\hat{\beta })=0$ [6]. *Then, given* z *and* $z^{*}$, *we have that*

(8) $$\begin{array}{ll} & n^{1/2}\{\hat{w}(t|z,z^{*})-w(t|z,z^{*})\}\\ & \;=n^{-1/2}\overset{n}{\underset{i=1}{\sum }}\left[\nabla \Omega_{\eta }(t;z,z^{*})\psi_{\eta }({𝒪}_{i})\right.\\ & \;-\left.2w(t|z,z^{*})\{1-\mu (z,z^{*};\beta )\}{\left(z-z^{*}\right)}^{T}A{\left(\beta \right)}^{-1}\kappa ({𝒪}_{i};\beta )\right]+o_{p}(1), \end{array}$$

*where*

$$\begin{array}{ll} \nabla \Omega_{\eta }(t;z,z^{*}) & =-\mu (z,z^{*};\beta )\left\{S(t|z^{*};\eta )\frac{\partial }{\partial \eta }S(t|z;\eta )\right.\\ & \;\left.+\;S(t|z;\eta )\frac{\partial }{\partial \eta }S(t|z^{*};\eta )\right\},\\ A(\beta ) & =-E\left[R_{ij}(\infty )\mu (Z_{i},Z_{j};\beta )\right.\\ & \;\times \left.\{1-\mu (Z_{i},Z_{j};\beta )\}{\left(Z_{i}-Z_{j}\right)}^{⊗2}\right],\\ \text{and}\;\kappa ({𝒪}_{i};\beta ) & =E\left\{(Z_{i}-Z_{j})M_{ij}(\infty |Z_{i},Z_{j};\beta )|{𝒪}_{i}\right\}. \end{array}$$

The PW model‐related A(β) and $\kappa ({𝒪}_{i};\beta )$ can always be estimated by

A^(β^)=−n2−1∑i=1n−1∑j=i+1nRij(∞)μ(Zi,Zj;β^)×{1−μ(Zi,Zj;β^)}(Zi−Zj)⊗2andκ^(𝒪i;β^)=(n−1)−1∑j≠i(Zi−Zj)Mij(∞|Zi,Zj;β^),

respectively [6]. Both $\nabla \Omega_{\eta }(t;z,z^{*})$ and $\psi_{\eta }({𝒪}_{i})$, however, depend on the choice of the TFE model.

For the TFE, write $\tilde{X}=\tilde{T}\wedge C$, $\tilde{\delta }=I(\tilde{T}\leq C)$, and $\tilde{N}(t)=I(\tilde{X}\leq t,\tilde{\delta }=1)$. Consider the Cox PH model

(9) $$\mathrm{pr}(t\leq \tilde{T}<t+dt|\tilde{T}\geq t;Z)=\exp(\gamma^{T}Z)d\Lambda_{0}(t),$$

where γ is regression parameter and $\Lambda_{0}$ is the baseline cumulative hazard function. In this case, $S(t|Z;\eta )=\exp\left\{-\exp(\gamma^{T}Z)\Lambda_{0}(t)\right\}$, with $\eta =(\gamma ,\Lambda_{0})$.

Corollary 1 *Under the Cox model* (9), *with* $\hat{\eta }=(\hat{\gamma },{\hat{\Lambda }}_{0})$ *denoting the standard partial‐likelihood/Breslow estimators, we can estimate* $\nabla \Omega_{\eta }(t;z,z^{*})\psi_{\eta }({𝒪}_{i})$ *by*

$$\begin{array}{ll} & \nabla {\hat{\Omega }}_{\eta }(t;z,z^{*}){\hat{\psi }}_{\eta }(𝒪)\\ & \;=\mu (z,z^{*};\hat{\beta })S(t|z;\hat{\eta })S(t|z^{*};\hat{\eta })\left[\left\{\hat{H}(t;z)+\hat{H}(t;z^{*})\right\}\right.\\ & \;\times {\hat{ℐ}}^{-1}\int_{0}^{\infty }\left\{Z-\hat{ℰ}(u)\right\}d\tilde{M}(u;\hat{\eta })\\ & \;\left.+\;\left\{\exp({\hat{\gamma }}^{T}z)+\exp({\hat{\gamma }}^{T}z^{*})\right\}\int_{0}^{t}{\hat{s}}^{\left(0\right)}{\left(u\right)}^{-1}d\tilde{M}(u;\hat{\eta })\right], \end{array}$$

*where*

$$\begin{array}{ll} \tilde{M}(t;\eta ) & =\tilde{N}(t)-\int_{0}^{t}I(\tilde{X}\geq u)\exp(\gamma^{T}Z)d\Lambda_{0}(u),\\ \hat{s^{\left(k\right)}}(u) & =n^{-1}\overset{n}{\underset{i=1}{\sum }}I({\tilde{X}}_{i}\geq u)\exp({\hat{\gamma }}^{T}Z_{i})Z_{i}^{⊗k}\;(k=0,1,2),\\ \hat{ℰ}(u) & ={\hat{s}}^{\left(1\right)}(u)/{\hat{s}}^{\left(0\right)}(u),\\ \;\hat{ℐ} & =\int_{0}^{\infty }\left\{\hat{s^{\left(2\right)}}(t)/\hat{s^{\left(0\right)}}(t)-\hat{ℰ}{\left(t\right)}^{⊗2}\right\}n^{-1}\overset{n}{\underset{i=1}{\sum }}d{\tilde{N}}_{i}(t),\\ \text{and}\;\hat{H}(t;z) & =\exp({\hat{\gamma }}^{T}z)\int_{0}^{t}\{z-\hat{ℰ}(u)\}d{\hat{\Lambda }}_{0}(u). \end{array}$$

The asymptotic linear expansion in Equation (8) means that $\hat{w}(t|z,z^{*})$ is approximately a Gaussian process, whose variance function can be estimated by 

(10)

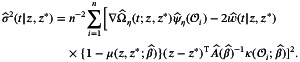




Since the estimand is constrained in [0,1], we recommend the logit transformation (i.e., logit(x)=log{x/(1−x)}) for construction of confidence intervals. Straightforwardly, the 100(1−α)% (pointwise) logit‐transformed confidence interval is

$$w(t|z,z^{*})\in \text{expit}\left[\text{logit}\{\hat{w}(t|z,z^{*})\}\pm z_{1-\alpha /2}{\hat{\sigma }}_{l}(t|z,z^{*})\right],$$

where expit(x)=exp(x)/{1+exp(x)}, ${\hat{\sigma }}_{l}(t|z,z^{*})={\left[\hat{w}(t|z,z^{*})\{1-\hat{w}(t|z,z^{*})\}\right]}^{-1}\hat{\sigma }(t|z,z^{*})$, and $z_{1-\alpha /2}$ is the 100(1−α/2)th percentile of the standard normal distribution.

### 2.4 Win Ratio, Win Odds, and Net Benefit

Given the estimated win and loss probabilities, we can now obtain the contrast measures:

(11) $$\begin{array}{ll} \text{Win ratio:}\;\text{WR}(t|z,z^{*}) & =\frac{w(t|z,z^{*})}{w(t|z^{*},z)}=\exp\left\{\beta^{T}(z-z^{*})\right\};\\ \text{Net benefit:}\;\text{NB}(t|z,z^{*}) & =w(t|z,z^{*})-w(t|z^{*},z)\\ \text{Win odds:}\;\text{WO}(t|z,z^{*}) & =\frac{\begin{array}{l} w(t|z,z^{*})\\ +0.5\{1-w(t|z,z^{*})-w(t|z^{*},z)\} \end{array}}{\begin{array}{l} w(t|z^{*},z)\\ +0.5\{1-w(t|z,z^{*})-w(t|z^{*},z)\} \end{array}}\\ & =\frac{1+\text{NB}(t|z,z^{*})}{1-\text{NB}(t|z,z^{*})}. \end{array}$$

The win ratio, a constant feature produced solely by the PW model, is the simplest to handle. Rather than substituting the win and loss probabilities, we estimate it directly by $\exp\left\{{\hat{\beta }}^{T}(z-z^{*})\right\}$, with confidence intervals derived from the variance matrix of $\hat{\beta }$. For the net benefit and win odds, plug‐in estimators apply. However, the correlations between the estimated win and loss probabilities need to be accounted for in making inferences.

Let ${\hat{\sigma }}_{12}(t|z,z^{*})$ denote the robust *covariance* estimator for $\hat{w}(t|z,z^{*})$ and $\hat{w}(t|z^{*},z)$, constructed similarly to (10). Straightforwardly, the standard error of the plug‐in $\hat{\text{NB}}(t|z,z^{*})$ is estimated by

$${\hat{\sigma }}_{\text{nb}}(t|z,z^{*})=\sqrt{{\hat{\sigma }}^{2}(t|z,z^{*})+{\hat{\sigma }}^{2}(t|z^{*},z)-2{\hat{\sigma }}_{12}(t|z,z^{*})}.$$

For confidence‐interval construction, it is customary to map the net benefit from its contrained range of [−1,1] onto ℝ. This can be done, e.g., by the inverse hyperbolic tangent function:

$$\text{arctanh}(x)=2^{-1}\log\left(\frac{1+x}{1-x}\right).$$

With $\tanh(x)={\text{arctanh}}^{-1}(x)=\{\exp(x)-\exp(-x)\}/\{\exp(x)+\exp(-x)\}$, and using the delta method, we obtain a 100(1−α)% confidence interval

(12) $$\begin{array}{ll} \text{NB}(t|z,z^{*}) & \in \tanh\left[\text{arctanh}\{\hat{\text{NB}}(t|z,z^{*})\}\;\pm \;z_{1-\alpha /2}\right.\\ & \;\times \left.{\left\{1-\hat{\text{NB}}{\left(t|z,z^{*}\right)}^{2}\right\}}^{-1}{\hat{\sigma }}_{\text{nb}}(t|z,z^{*})\right]. \end{array}$$

The arctanh transformation also simplifies the case with the win odds, which can be expressed as $\text{WO}(t|z,z^{*})=\exp\left[2\text{arctanh}\{\text{NB}(t|z,z^{*})\}\right]$. As a result, its 100(1−α)% confidence interval is

(13) $$\begin{array}{cc} \text{WO}(t|z,z^{*}) & \in \exp\left[2\text{arctanh}\{\hat{\text{NB}}(t|z,z^{*})\}\;\right.\\ & \;\pm \left.2z_{1-\alpha /2}{\left\{1-\hat{\text{NB}}{\left(t|z,z^{*}\right)}^{2}\right\}}^{-1}{\hat{\sigma }}_{\text{nb}}(t|z,z^{*})\right]. \end{array}$$

The arctanh and tanh transformations are conveniently implemented in base R's atanh() and tanh() functions, respectively. The resulting confidence intervals in Equations (12) and (13) are always contained in [−1,1] and [0,∞), the proper ranges of the net benefit and win odds, respectively. This approach also saves computation and ensures consistency in the coverage of both quantities, given their direct link to each other.

### 2.5 Model Diagnostics and Possible Stratification

For reliable predictions, it is important that both the PW and TFE models fit the data reasonably well. Particular attention should be paid to the functional forms of quantitative covariates, as their default forms may not be appropriate.

To check the form of the k covariate $Z_{\cdot k}$ in PW model (3), construct a plot of

(14) $${\hat{M}}_{i}^{\left(n\right)}\equiv {\left(n-1\right)}^{-1}\underset{j\neq i}{\sum }M_{ij}(\infty |Z_{i},Z_{j};\hat{\beta })\;\text{vs.}\;Z_{ik}\;(i=1,\ldots ,n)$$

where $Z_{ik}$ is $Z_{\cdot k}$ on the ith subject. Here, the evaluation time ∞ means that all observed events up to the maximum follow‐up are accounted for. Hence, the residual ${\hat{M}}_{i}^{\left(n\right)}$ can be interpreted as the observed minus the model‐predicted proportion of wins for the ith subject. For that reason, it is called the subject‐specific win residual. Rationales for using this residual in model diagnostics are detailed in a separate paper. Briefly, we expect the residuals to be randomly scattered around zero without any pattern if the form of $Z_{\cdot k}$ is correct. Otherwise, we may need to transform $Z_{\cdot k}$, by grouping or spline smoothing, to account for possible nonlinearities in its effect. Similar techniques apply to the Cox model by replacing ${\hat{M}}_{i}^{\left(n\right)}$ in Equation (14) with the standard martingale residual. An example is offered in Section 4 for the HF‐ACTION trial.

Another important set of assumptions concerns proportionality. For the Cox model, this means proportionality of hazard rates and can be checked using Schoenfeld residuals [15] (which can be obtained from cox.zph() in the survival R‐package). For the PW model, however, this means proportionality of win‐loss probabilities, i.e., the right hand side of (3) is independent of t. This can be checked by plotting a standardized version of the “score residuals”

U^(t)=n2−1∑i=1n−1∑j=i+1n(Zi−Zj)Mij(t|Zi,Zj;β^)

over t, which is implemented in the existing WR package.

When proportionality is in doubt for a categorical covariate, whether in the PH or PW models, a common strategy is to stratify the model by its levels. The idea is to narrow the proportionality requirement to subjects within each stratum while allowing nonproportionality across strata. Fortunately, both models support this feature.

In addition to {Y(X),X,Z}, let 𝒮=1,…,L denote the stratum affiliation of a generic subject, where L is the total number of strata. Then, the win‐loss probabilities within the lth stratum (l=1,…,L) are defined by

(15) $$w_{l}(t;z,z^{*})=\mathrm{pr}\{𝒲(Y_{i},Y_{j})(t)=1|Z_{i}=z,Z_{j}=z^{*},{𝒮}_{i}={𝒮}_{j}=l\}.$$

To estimate (15), we only need a stratified PW model [16]

$$\frac{w_{l}(t|Z_{i},Z_{j})}{w_{l}(t|Z_{j},Z_{j})}=\exp\{\beta^{T}(Z_{i}-Z_{j})\},$$

and stratified Cox TFE model $\mathrm{pr}\{\tilde{T}>t|Z,𝒮=l\}=\exp\{-\exp(\gamma^{T}Z)\Lambda_{0l}(t)\}=:S(t|Z;\eta_{l})$, where $\Lambda_{0l}(t)$ is the stratum‐specific baseline function and $\eta_{l}=(\gamma ,\Lambda_{0l})$. For the two stratified models, the covariate effects β and γ are shared across the strata, but the strata effects are left nonparametric.

Let $\hat{\beta }$ and ${\hat{\eta }}_{l}$ denote the standard estimators of β and $\eta_{l}$ under the stratified PW and Cox models, respectively. Similarly to (7), we can estimate (15) by

$${\hat{w}}_{l}(t|z,z^{*})=\{1-S(t|z;{\hat{\eta }}_{l})S(t|z^{*};{\hat{\eta }}_{l})\}\frac{\exp\{{\hat{\beta }}^{T}z\}}{\exp\{{\hat{\beta }}^{T}z\}+\exp\{{\hat{\beta }}^{T}z^{*}\}}.$$

Asymptotic variance estimation along the lines of Proposition 1 and Corollary 1 is detailed in Section S1.2 of the Supporting Information.

## 3 Simulation Studies

The WR package, available on GitHub and CRAN, has been updated to incorporate the prediction tools introduced in this paper. After fitting a PW model using the standard pwreg() function, one can use the S3 method predict() on the fitted model object, along with two self‐provided covariate vectors, to compute the predicted win‐loss probabilities and, optionally, the win ratio/odds and net benefit measures over time. A code example is provided in the Appendix.

We conducted simulations to evaluate the performance of the prediction procedures. Consider the common scenario of bivariate outcomes (D,T), with D prioritized over T as formulated in Equation (1). Let $Z={\left(Z_{\cdot 1},Z_{\cdot 2},Z_{\cdot 3}\right)}^{T}$, with $Z_{\cdot 1}∼N(0,1)$, $Z_{\cdot 1}∼N(-1,1)$, and $Z_{\cdot 3}∼\text{Bernoulli}(0.5)$. Given the covariates, we generated the outcomes through the Gumbel–Hougaard copula model [17]

$$\begin{array}{ll} \mathrm{pr}(D>s,T>t|Z) & =\exp\left(-\left[{\left\{\exp(-\beta^{T}Z)\lambda_{D}s\right\}}^{\kappa }\right.\right.\\ & \;\left.{\left.+{\left\{\exp(-\beta^{T}Z)\lambda_{H}t\right\}}^{\kappa }\right]}^{1/\kappa }\right). \end{array}$$

This model is attractive because it simultaneously satisfies the PW model (3) with regression parameter β and the Cox model (9) for the TFE with γ=−β and $\Lambda_{0}(t)={\left(\lambda_{D}^{\kappa }+\lambda_{H}^{\kappa }\right)}^{1/\kappa }t$. We set $\beta ={\left(0.5,0,-0.5\right)}^{T}$, $\lambda_{D}=0.1$, $\lambda_{H}=1$, κ=2 (leading to Kendall's rank correlation of $1-\kappa^{-1}=50\%$ between D and T [17]). For censoring, set C∼Un[0.2,4]∧Expn(0.02). With maximum follow‐up τ=4, these configurations give rise to a death rate of about 25% and a nonfatal event rate of about 75%.

With sample size n=100,200,500,1000 and 2000, we generated N=2000 replicate datasets and fit the PW model to each. As shown in Table S1 of the Supporting Information, the estimator $\hat{\beta }$ is practically unbiased, with accurately estimated standard errors and well‐calibrated confidence intervals. These results validate the correct fitting of the PW models.

Next, based on the fitted PW model for each dataset, we predicted the win‐loss probabilities for two pairs of covariate vectors:

- Pair 1: $z={\left(1,0,0\right)}^{T}$ vs. $z^{*}={\left(0,0,0\right)}^{T}$;
- Pair 2: $z={\left(0,0,0\right)}^{T}$ vs. $z^{*}={\left(-1,0,0\right)}^{T}$.

**FIGURE 1:**
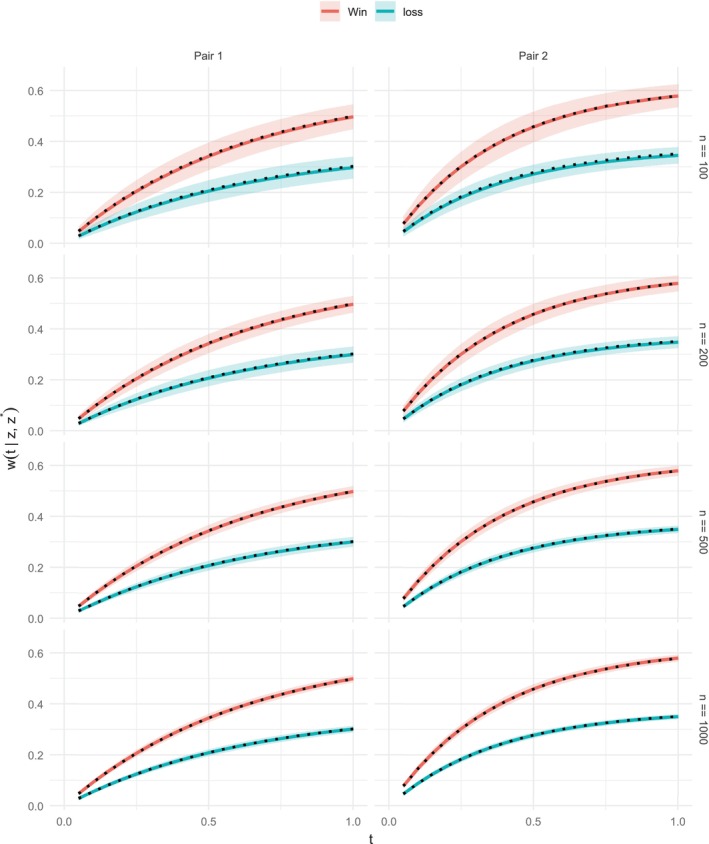
Simulation results for the estimation of $w(t|z,z^{*})$. Solid lines and shaded areas: Average estimates ± standard deviations across N=2000 samples; dotted lines: true values.

This amounts to an assessment of the effects of increasing $Z_{\cdot 1}$ by 1 (standard deviation) from a baseline of 0 and 1, respectively, while holding the other two covariates constant at $Z_{\cdot 2}=Z_{\cdot 3}=0$. Figure 1 shows the average curves of $\hat{w}(t|z,z^{*})$ and $\hat{w}(t|z^{*},z)$ for Pairs 1 and 2 along with their standard deviations. The estimates align remarkably well with the true curves, even for a sample size as small as n=100. The uncertainties around the estimates are also reasonably small, reflecting the efficiency of parsimonious modeling provided by the PW and Cox models.

To evaluate the variance estimator for $\hat{w}(t|z,z^{*})$, we examined the inferential results at t=0.05,0.1,1, and 4, representing short‐ to long‐term evaluation times within [0,4]. As summarized in Table 1, not only are the estimates virtually unbiased, the standard error estimators and confidence intervals also provide accurate measures of uncertainty. This suggests that the robust approach to asymptotic variance estimation, detailed in Proposition 1 and Corollary 1, performs well in finite samples. The results for the loss probability $w(t|z^{*},z)$ show similar patterns and are presented in Table S2 of the Supporting Information.

**TABLE 1:** Estimation and inference of $w(t|z,z^{*})$ at specific t's.

n	t	Pair 1					Pair 2				
		True	Bias	SE	SEE	CP	True	Bias	SE	SEE	CP
100	0.05	0.048	−0.002	0.019	0.019	0.965	0.078	−0.002	0.031	0.030	0.964
	0.10	0.093	−0.002	0.028	0.028	0.959	0.145	−0.002	0.043	0.042	0.955
	1.00	0.499	−0.002	0.049	0.048	0.943	0.579	0.000	0.045	0.045	0.953
	4.00	0.621	−0.002	0.034	0.034	0.945	0.622	0.003	0.034	0.034	0.949
200	0.05	0.048	0.000	0.014	0.014	0.950	0.078	0.000	0.022	0.021	0.949
	0.10	0.093	−0.001	0.020	0.020	0.951	0.145	0.000	0.030	0.030	0.957
	1.00	0.499	−0.002	0.033	0.033	0.956	0.579	0.000	0.031	0.031	0.949
	4.00	0.621	−0.001	0.024	0.024	0.941	0.622	0.002	0.024	0.024	0.938
500	0.05	0.048	0.000	0.009	0.009	0.963	0.078	0.000	0.013	0.014	0.957
	0.10	0.093	0.000	0.013	0.013	0.946	0.145	0.000	0.019	0.019	0.949
	1.00	0.499	−0.001	0.021	0.021	0.942	0.579	0.000	0.020	0.019	0.942
	4.00	0.621	0.000	0.015	0.015	0.944	0.622	0.001	0.015	0.015	0.944
1000	0.05	0.048	0.000	0.006	0.006	0.951	0.078	0.000	0.010	0.010	0.951
	0.10	0.093	0.000	0.009	0.009	0.952	0.145	0.000	0.013	0.013	0.947
	1.00	0.499	0.000	0.014	0.015	0.955	0.579	0.001	0.014	0.014	0.956
	4.00	0.621	0.000	0.010	0.010	0.953	0.622	0.001	0.010	0.010	0.951

*Note*: Each scenario is based on 2000 replicates.

Abbreviations: CP, empirical coverage probability of 95% confidence interval; SE, empirical standard deviation of estimator; SEE, empirical mean of standard error estimator.

Finally, we turned our attention to the contrast measures. Since the win ratio is constant and already covered in the PW modeling results in Table S1, we focused on the net benefit and win odds. Table 2 summarizes the estimation and inference of NB$\left(t|z,z^{*}\right)$ and WO$\left(t|z,z^{*}\right)$ across various time points and sample sizes. The low bias of the estimates is expected, as they are mere pointwise functions of the win and loss probabilities. The accurate coverage probabilities of the confidence intervals, however, indicate that the correlations between the win and loss probabilities are properly accounted for by the approach described in Section 2.4.

**TABLE 2:** Estimation and inference of time‐dependent net benefit and win odds.

		$z=z_{1},z^{*}=z_{2}$					$z=z_{2},z^{*}=z_{3}$				
n	t	NB$\left(t|z,z^{*}\right)$		WO$\left(t|z,z^{*}\right)$			NB$\left(t|z,z^{*}\right)$		WO$\left(t|z,z^{*}\right)$		
		True	Bias	True	Bias	CP	True	Bias	True	Bias	CP
100	0.1	0.037	0.000	1.076	−0.001	0.941	0.057	0.001	1.121	0.004	0.939
	0.5	0.136	−0.001	1.314	0.006	0.958	0.180	0.004	1.440	0.025	0.951
	1.0	0.196	0.003	1.488	0.020	0.957	0.228	0.006	1.590	0.040	0.946
	4.0	0.245	0.005	1.647	0.040	0.945	0.245	0.007	1.649	0.039	0.946
200	0.1	0.037	0.000	1.076	0.000	0.954	0.057	0.001	1.121	0.002	0.948
	0.5	0.136	0.000	1.314	0.002	0.962	0.180	0.002	1.440	0.012	0.952
	1.0	0.196	−0.001	1.488	0.009	0.949	0.228	0.003	1.590	0.020	0.942
	4.0	0.245	0.003	1.647	0.021	0.938	0.245	0.004	1.649	0.025	0.935
500	0.1	0.037	0.000	1.076	0.000	0.949	0.057	0.000	1.121	0.001	0.949
	0.5	0.136	0.000	1.314	0.002	0.953	0.180	0.001	1.440	0.006	0.950
	1.0	0.196	0.001	1.488	0.006	0.949	0.228	0.002	1.590	0.011	0.945
	4.0	0.245	−0.001	1.647	0.011	0.943	0.245	0.003	1.649	0.013	0.945
1000	0.1	0.037	0.000	1.076	0.000	0.952	0.057	0.001	1.121	0.001	0.952
	0.5	0.136	0.001	1.314	0.002	0.952	0.180	0.001	1.440	0.005	0.955
	1.0	0.196	0.001	1.488	0.005	0.955	0.228	0.002	1.590	0.007	0.955
	4.0	0.245	−0.002	1.647	0.007	0.952	0.245	0.002	1.649	0.008	0.950

*Note*: See to Table 1. Net benefit and win odds share the same coverage probability as their confidence intervals (12) and (13) are consistent.

In sum, the findings from these simulation studies suggest that both pointwise predictions and their uncertainty quantifications perform well in practical settings. Furthermore, the parsimony of the PW‐TFE dual models offer the added advantage of stabilizing estimates across the time spectrum, particularly at time points where events are sparse.

On the other hand, to assess the impact of model misspecification, we also examined scenarios in which the PW and Cox TFE models are jointly violated. This is achieved by using different values of β in the first display of Section 3 for D and T. Under otherwise the same set‐up, the coefficient of $Z_{\cdot 1}$ is set to 0.5 for death and 0.1 for the nonfatal event, corresponding to the differential hazard ratios of 1.65 and 1.10, respectively. In this case, the PW assumption is clearly violated, as shown by the cumulative win score residuals in the right panel below (which suggest that the win ratio is increasing over time; see Mao and Wang [6]). The PH assumption for the TFE is also violated, though this is less apparent from the Schoenfeld residuals because the TFE is dominated by nonfatal events and therefore less sensitive to the effect on death.

**FIGURE 2:**
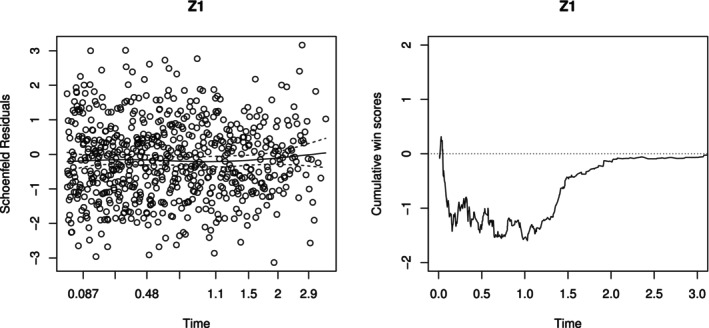
Residual analyses under a misspecified model. Left: Schoenfeld residuals for the TFE Cox model; right: cumulative win score residuals for the PW model.

**FIGURE 3:**
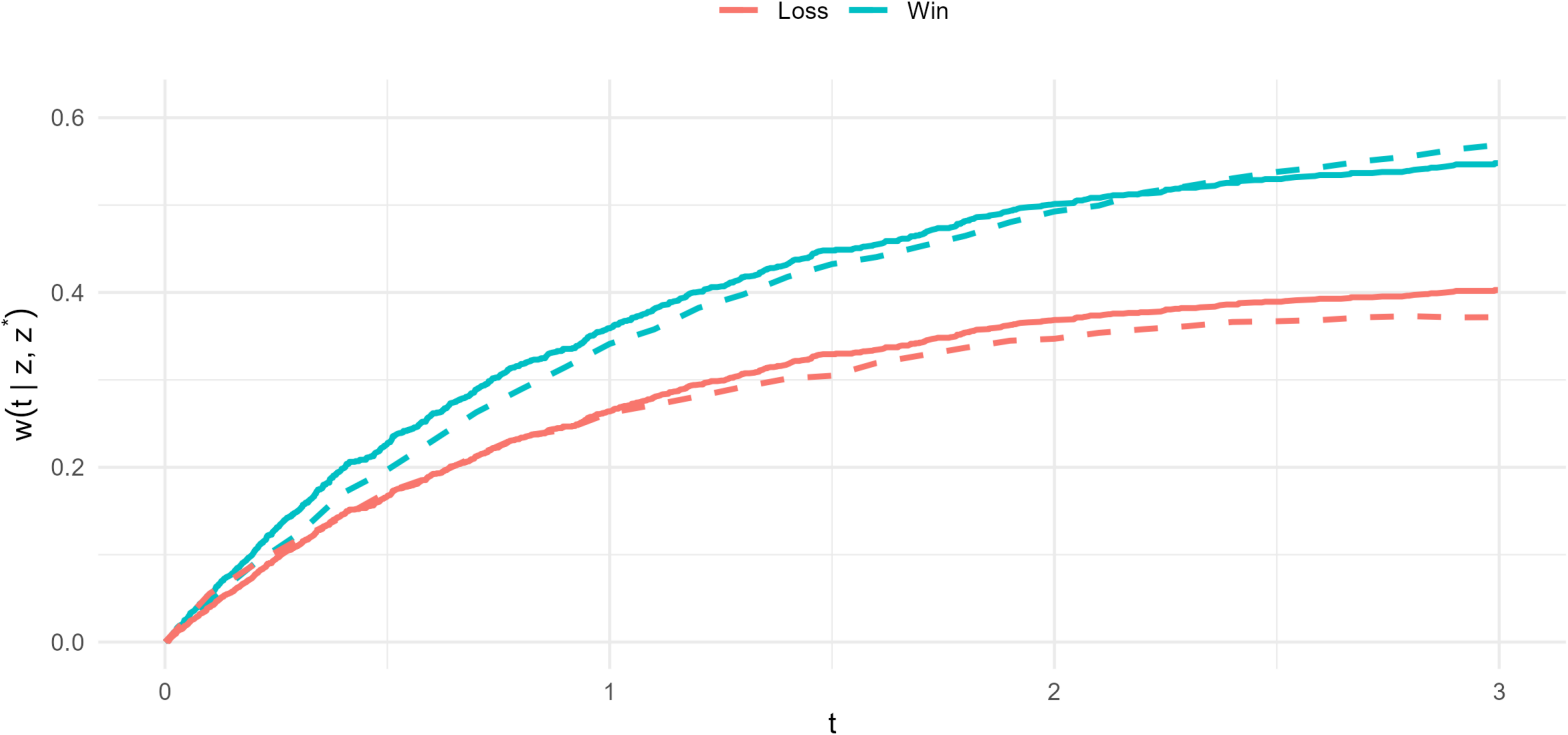
Estimated vs. true win/loss probabilities over time under a misspecified PW model. Solid: Estimates based on sample size n=1000; dashed: true values.

Under this misspecified model, with n=1000, there is clear bias between the estimated win/loss probabilities (solid) and the true values (dashed), as shown in Figure 3 below. This contrasts with Figure 1 of the main text, where model assumptions are satisfied. This example highlights the limitations of existing models and underscores the importance of model checking before using their results (Figures 2 and 3).

## 4 A Real Example

The HF‐ACTION (Heart Failure: A Controlled Trial Investigating Outcomes of Exercise Training) study is one of the largest randomized controlled trials designed to evaluate the effects of aerobic exercise training in patients with chronic heart failure (HF) and reduced ejection fraction (HFrEF) [18]. Conducted across 82 centers in the United States, Canada, and France, the trial enrolled 2331 medically stable HF patients from 2003 to 2007. Participants were randomized to receive either usual care plus a structured exercise training program or usual care alone, and followed over a median length of 30 months. The primary endpoint was time to the first occurrence of all‐cause mortality and all‐cause hospitalization. Secondary endpoints included cardiovascular mortality or hospitalization, and quality of life improvements. Initial findings showed that exercise training resulted in a modest reduction in the primary endpoint, with a hazard ratio of 0.93 and a p‐value of 0.13 [18].

In addition, the primary analysis demonstrated that baseline CPX duration, the total time a patient is able to perform a cardiopulmonary exercise test before experiencing discomfort, strongly correlates with patient outcomes regardless of treatment assignment. This finding is supported by other studies pointing to the remarkable prognostic value of CPX parameters [19, 20, 21, 22].

As an illustration, we analyze the HF‐ACTION data using the PW model, which prioritizes death over hospitalization. Our focus is on evaluating the win‐loss probabilities as baseline CPX duration varies across the patient population. To do so, we adjust for a number of key baseline risk factors, as tabulated in Table 3 by treatment arm and overall (n=2130). These include patient age, sex, race, HF etiology (ischemic or non‐ischemic), body mass index (BMI), and NYHA (New York Heart Association) functional classification (II–IV), six‐minute walk distance (6MWD), left ventricular ejection fraction (LVEF), as well as CPX duration.

**TABLE 3:** Baseline characteristics and outcome summary for the HF‐ACTION study cohort.

Characteristic	Overall, n=2130	Usual Care, N=1070	Training, N=1060
Age (years)	59 (51, 68)	59 (51, 68)	59 (51, 67)
Sex			
Male	1531 (72%)	789 (74%)	742 (70%)
Female	599 (28%)	281 (26%)	318 (30%)
Race			
Non‐white	808 (38%)	394 (37%)	414 (39%)
White	1322 (62%)	676 (63%)	646 (61%)
Etiology			
Ischemic	1096 (51%)	546 (51%)	550 (52%)
Non‐ischemic	1034 (49%)	524 (49%)	510 (48%)
Body Mass Index	30 (26, 35)	30 (26, 35)	30 (26, 35)
Unknown	5	3	2
NYHA class			
II	1351 (63%)	691 (65%)	660 (62%)
III	759 (36%)	371 (35%)	388 (37%)
IV	20 (0.9%)	8 (0.7%)	12 (1.1%)
CPX duration (min)	9.7 (7.0, 12.0)	9.7 (7.0, 12.1)	9.6 (6.9, 12.0)
Unknown	20	11	9
6MWD (meters)	372 (300, 436)	375 (300, 435)	366 (299, 436)
Unknown	46	24	22
LVEF (%)	25 (20, 30)	25 (20, 30)	25 (20, 30)
Unknown	4	1	3
Death (Outcome)	351 (16%)	183 (17%)	168 (16%)
Hospitalization (Outcome)	1357 (64%)	696 (65%)	661 (62%)

*Note*: Median (Inter‐quartile range); Frequency (%).

**FIGURE 4:**
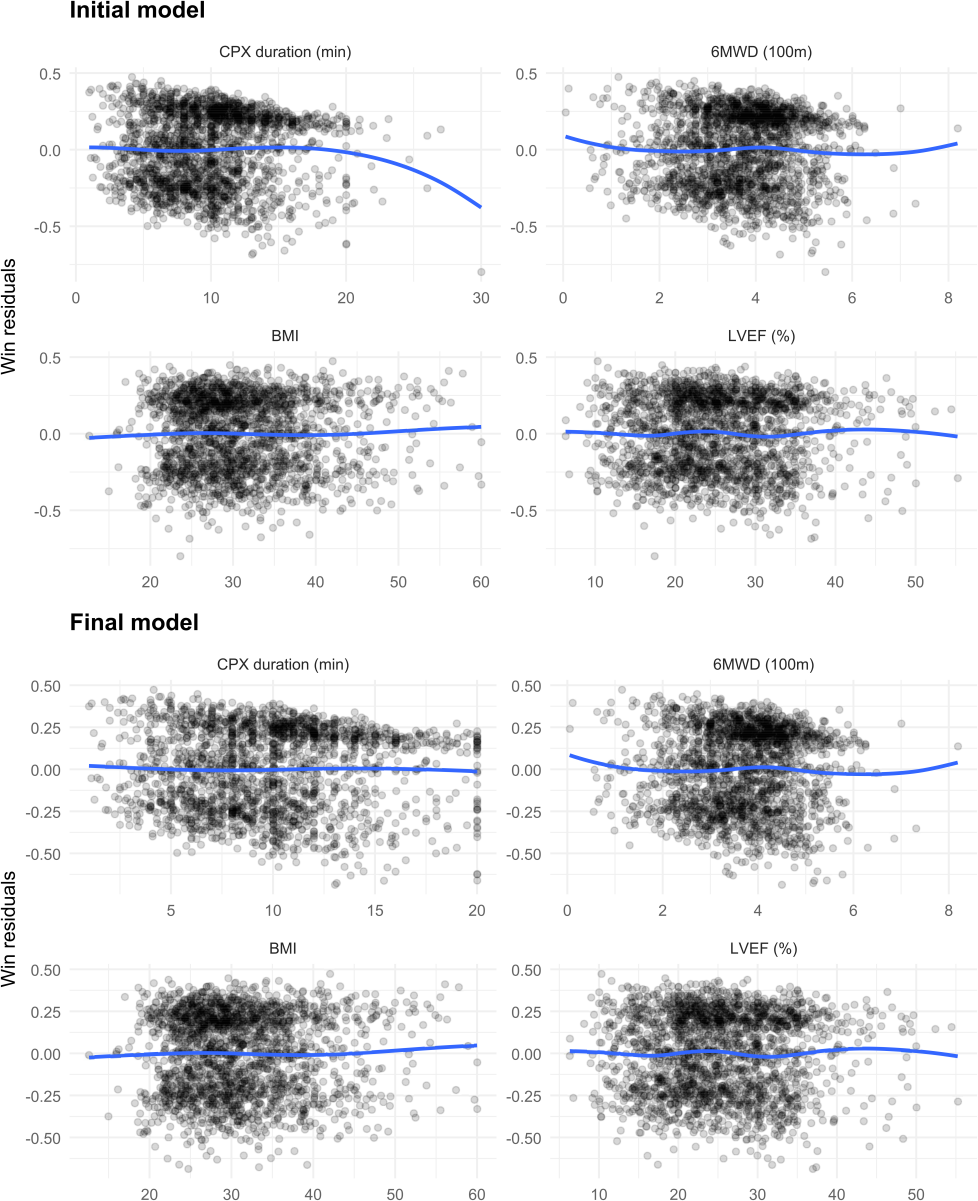
Residual analysis for initial and final PW models. The latter addresses the nonlinear effect of CPX duration beyond 20 min.

Over 4.2 years of follow‐up, among all paired comparisons within the cohort, 82.9% produce a determinate result (a win and a loss), including 24.2% on death and the remaining 58.7% on hospitalization. For regression analysis, suppose that a prespecified list of covariates includes age, sex, race, and HF etiology BMI, CPX duration, NYHA class, 6MWD, and LVEF. As a first step, we fit an initial PW model with all continuous covariates (CPX duration, 6MWD, BMI, LVEF) entered in linear forms. Score processes from the PW model and the Cox model for the TFE indicates that the proportionality assumptions are reasonable for both models. However, a residual analysis based on (14) for the continuous covariates reveals a potentially misspecified form of CPX duration (see the first part of Figure 4). Specifically, while the win residuals remain roughly patternless over the range of 0–20 min, there is a clear downward shift beyond 20 min. Since ${\hat{M}}_{i}^{\left(n\right)}$ is the difference between empirical and model‐predicted win proportions, their values trending negative means there are fewer wins than predicted in that region. A likely explanation for this deficit is that the beneficial effect of longer CPX duration has plateaued at around 20 min, and further increases beyond this mark fail to produce the same benefits predicted on a linear trend. This is also the likely cause of a similar feature in the martingale residuals of the Cox model (Figure S1 of Supporting Information).

To account for this ceiling effect, we apply a threshold of 20 min to CPX duration, essentially assigning a value of 20 to all values longer than 20 min. After refitting the model with this transformation, residual analysis indicates that the nonlinearity has been adequately addressed (second part of Figure 4). The results of the final model are summarized in Table 4. (A corresponding Cox model for the TFE is fitted and summarized in the Supporting Information, showing covariate effects consistent with those from the PW model.) Notably, a one‐minute increase in CPX duration leads to a win ratio of 1.09, representing a remarkable 9% increase in the likelihood of a more favorable outcome. While the residual plot (Figure 4) confirms that this relative effect holds across the entire range of 0–20+ min, the model provides no information on the absolute win and loss probabilities. These depend on the event rates that are affected by the time frame and the base level of CPX duration as well as its increment.

**TABLE 4:** Final PW modeling of the HF‐ACTION study.

	WR	95% CI	p
Training vs. UC	1.07	[0.96, 1.20]	0.233
Age (years)	1.00	[1.00, 1.01]	0.597
Sex			
Male	—	—	—
Female	1.40	[1.22, 1.60]	<0.001
Race			
White	—	—	—
Non‐white	0.84	[0.74, 0.95]	0.007
Etiology			
Non‐ischemic	—	—	—
Ischemic	0.87	[0.77, 0.99]	0.029
BMI	1.01	[1.00, 1.02]	0.010
CPX duration (min)	1.09	[1.06, 1.11]	<0.001
NYHA class			
II	—	—	—
III	0.80	[0.70, 0.91]	<0.001
IV	0.59	[0.36, 0.97]	0.039
6MWD (100m)	1.11	[1.04, 1.20]	0.003
LVEF (%)	1.02	[1.01, 1.03]	<0.001

Abbreviations: CI, confidence interval; UC, usual care; WR, win ratio.

**FIGURE 5:**
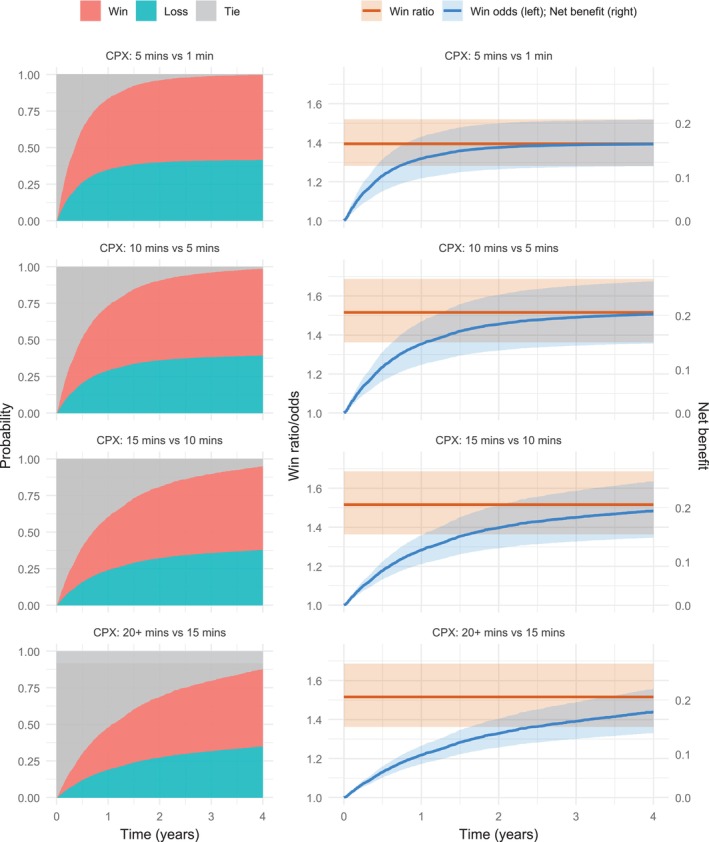
Predicted win‐loss probabilities and contrast measures over time comparing different CPX durations for a non‐white, non‐ischemic female patient of median age (59), receiving usual care, with a median BMI (30), median 6MWD (372m), and median LVEF (25%), under the final PW model for the HF‐ACTION study. Shaded area: 95% confidence interval.

For this additional information, we use the prediction tools to characterize the win and loss trajectories as CPX duration increases from 1 min (the cohort minimum) to 20+ min through several intermediate points. We perform this prediction for a non‐white, non‐ischemic female patient of median age (59), receiving usual care, with a median BMI (30), median 6MWD (372m), and median LVEF (25%). The left column of Figure 5 displays the time‐dependent probabilities of win, loss, and tie associated with 4‐ or 5‐minute increases in CPX (corresponding estimates based on categorized CPX are presented in the Supporting Information and are closely aligned with those obtained here under the continuous specification). The right column shows the corresponding trajectories of the win ratio (constant), win odds and net benefit (the latter two are directly linked by (11) and are represented by the same line, albeit on different y‐axis scales), along with their 95% confidence intervals.

When CPX duration is at the lower end of the spectrum, patients face a higher risk of death or hospitalization, resulting in elevated win and loss probabilities due to the lower likelihood of a tie. As CPX duration increases, however, fewer events are available for comparison, leading to a decline in the magnitude of both win and loss probabilities. Consequently, while the same increment in the predictor is associated with a constant relative change in win‐loss probability across the range, its impact on the absolute scale diminishes as the baseline value increases. This pattern is evident from a vertical comparison of the panels in Figure 5. For instance, when CPX duration increases from 5 to 10 min, the net benefit reaches 0.20 by year 3. However, when it increases from 15 to 20+ min, the net benefit is only 0.16 over the same time frame, despite the consistent win ratio of $1.09^{5}\approx 1.52$. For the reader's reference, the numerical values of the plotted metrics at years 1, 2, 3, and 4 are provided in Table S ofthe Supporting Information.

These findings highlight that, although the relative effect of CPX duration as summarized by the win ratio remains constant, the corresponding absolute benefit—as reflected by the win–loss probabilities and net benefit—depends strongly on baseline risk and follow‐up time. This illustrates the added value of probability‐based summaries for clinical interpretation.

## 5 Discussion and Extensions

The win ratio provides a succinct summary of treatment effects on a composite endpoint. As a relative measure, however, it can hide important context. When ties are predominant due to low event rates, the win ratio may exaggerate the effect size, highlighting the need for separate examinations of win and loss probabilities. This is particularly important in regression, where the win‐loss probabilities not only change over time but also depend on both the base values and increments of covariates. We have proposed a simple solution by supplementing the PW model, a parsimonious win ratio regression framework, with a time‐to‐first‐event (TFE) model (e.g., Cox model) that is minimally sufficient to infer the win‐loss trajectories for any given pair of covariate vectors. Its implementation in the open‐access WR package, along with the model‐checking tools, can help analysts build more reliable models and gain a deeper understanding of their results.

Some theoretical aspects of this approach remain of interest. One is the compatibility between the PW model and the chosen TFE model. In the simulation studies presented in Section 3, we used a model that simultaneously generates the PW model for the hierarchical composite and the Cox model for the TFE. Similarly, this compatibility holds for other Lehmann‐type models, as alluded to in Oakes [13].

However, it is not guaranteed that a PW model can be seamlessly paired with any TFE model, given the overlap in the endpoints being modeled. As a toy example, if the PW model is based solely on the TFE, our earlier work has demonstrated that it becomes equivalent to the Cox model. Consequently, any non‐Cox model for the TFE would be inappropriate. More generally, the more heavily the PW model draws on the TFE, the stronger the constraints on the model choice for the latter.

Therefore, model checking is essential to ensure that the chosen model fits the data reasonably well. Besides the Cox model, one can choose from a wide range of alternatives, such as the AFT or proportional odds models, whichever achieves the best fit. As shown in Equation (7), the TFE model affects the point estimates of win‐loss probabilities only through their implied survival functions, although the standard errors will need to be re‐derived for each specific model.

In addition to model choice, model specifications also enjoy greater flexibility than we have utilized throughout the paper. For notational simplicity, we have assumed that the same set of covariates Z are used in both PW and TFE models. Methodologically, this arrangement is nonessential. Different covariates, or the same covariates in different forms, can be used for each model. For example, one can use different smoothing splines for a continuous covariate if it fits differently in the PW and TFE models.

On the other hand, the current framework is heavily dependent on the PW model, which, by itself and in conjunction with a TFE model, places strong constraints on the evolution of win–loss probabilities over time. As the simulations show, considerable bias can result when these temporal constraints are violated. If the primary goal is prediction rather than inference under the PW model, one can instead model the win–loss probabilities directly without assuming they follow a particular pattern.

For example, at a given time t, the status of a win, loss, or tie may be treated as a multinomial response. A multinomial regression with tie as the reference category can then be specified as

(16) $$g\left\{\frac{w(t|Z_{i},Z_{j})}{\nu (t|Z_{i},Z_{j})}\right\}=\alpha (t)+\beta_{w}{\left(t\right)}^{T}Z_{i}+\beta_{l}{\left(t\right)}^{T}Z_{j},$$

where g(·) is a known link function and $\left\{\alpha (t),\beta_{w}(t),\beta_{l}(t)\right\}$ are unknown (time‐dependent) regression parameters. Under g(x)=log(x), the log–win ratio becomes

$$\log\{r(t|Z_{i},Z_{j})\}={\left\{\beta_{w}(t)-\beta_{l}(t)\right\}}^{T}(Z_{i}-Z_{j}).$$

Comparing this with (3), it is clear that the PW model is a special case of (16) with $\beta_{w}(t)-\beta_{l}(t)\equiv \beta$.

In general, the win‐loss probabilities for any given pair of covariates can be obtained directly from Equation (16), provided the regression parameters have been estimated. In the Supporting Information, we explore extending the inverse probability censoring weighting (IPCW) approach of Dong et al. [23] from a two‐sample setting to estimation under (16), without parameterizing or smoothing $\left\{\alpha (t),\beta_{w}(t),\beta_{l}(t)\right\}$ over t. Results from these more flexible models can then be compared with those obtained under the current method, such as the win–loss probabilities plotted in Figure 5, as a diagnostic tool for the latter.

## Funding

This work was supported by the National Heart, Lung, and Blood Institute (Grant No. R01HL149875).

## Conflicts of Interest

The author declares no conflicts of interest.

## Supporting information




**Data S1**: Web Appendices, tables, and figures referenced in the text can be found in the Supporting Information section at the end of the article.

**Data S2**: sim70569‐sup‐0002‐Supinfo.zip.

## Data Availability

The data that support the findings in this article are subject to a Research Materials Distribution Agreement (RMDA) with the Biologic Specimen and Data Repository Information Coordinating Center (BioLINCC) of the National Heart, Lung, and Blood Institute (NHLBI). They are not publicly available due to privacy or ethical restrictions.

## Appendix A

### A Code Example Using the WR Package

A simple reproducible code example is provided below.

As can be seen from above, predict() returns a tibble with the following columns:

- time: time vector t;
- win, win_se, win_high, win_low: win probability $\hat{w}(t|z_{1},z_{2})$, its standard error, upper and lower 95% confidence limits;
- loss, loss_se, loss_high, loss_low: loss probability $\hat{w}(t|z_{2},z_{1})$, its standard error, upper and lower 95% confidence limits;
  Below are the additional output requested by constrast = TRUE:
- wr, wr_low, wr_high: (time‐constant) win ratio $\hat{w}(t|z_{1},z_{2})/\hat{w}(t|z_{2},z_{1})$ and its 95% confidence limits;
- nb, nb_low, nb_high: net benefit $\hat{w}(t|z_{1},z_{2})-\hat{w}(t|z_{2},z_{1})$ and its 95% confidence limits;
- wo, wo_low, wo_high: win odds $\left\{1+\hat{\text{NB}}(t|z_{1},z_{2})\}/\{1-\hat{\text{NB}}(t|z_{1},z_{2})\right\}$ and its 95% confidence limits;
- nb_wo_z: z‐statistic for testing net benefit or win odds (same due to their direct relationship), which can be used to calculate the p‐value.
