# Supplementary material for: Predicting Win‐Loss Probabilities for Composite Time‐to‐Event Outcomes Under The Proportional Win‐Fractions Regression Model
Source: Stat Med. 2026 Apr 28;45:e70569. doi: 10.1002/sim.70569 (PMC13124461; doi:10.1002/sim.70569)
Supplement: Supplementary file 1 — Data S1: Web Appendices, tables, and figures referenced in the text can be found in the Supporting Information section at the end of the article. [file SIM-45-0-s002.pdf]

Received: Added at production | Revised: Added at production | Accepted: Added at production
DOI: xxx/xxxx

STATISTICS IN MEDICINE

Supporting Information for "Predicting win-loss probabilities for composite time-to-event outcomes under the proportional win-fractions regression model"

Lu Mao

[1]Department of Biostatistics and Medical Informatics, School of Medicine and Public Health, University of Wisconsin-Madison, Madison, Wisconsin, USA

Correspondence
*Department of Biostatistics and Medical Informatics, School of Medicine and Public Health, University of Wisconsin-Madison, Madison, WI 53726
Email: lmao@biostat.wisc.edu

Summary

This web appendix contains technical results and additional numerical studies referenced throughout the main text.

S1 | TECHNICAL RESULTS

S1.1 | Proof of Corollary 1

As the maximum partial-likelihood estimator, $\widehat\gamma$ solves

$$n^{-1}\sum_{i=1}^n\int_0^\infty\left\{Z_i-\frac{\sum_{j=1}^n I(\widetilde X_j\geq t)\exp(\widehat\gamma^{\mathrm T}Z_j)Z_j}{\sum_{j=1}^n I(\widetilde X_j\geq t)\exp(\widehat\gamma^{\mathrm T}Z_j)}\right\}\mathrm d\widetilde N_i(t)=0.$$

By standard results, we have that

$$n^{1/2}(\widehat\gamma-\gamma)=n^{-1/2}\sum_{i=1}^n\mathcal I^{-1}\int_0^\infty\{Z_i-\mathcal E(t)\}\mathrm d\widetilde M_i(t;\eta)+o_p(1), \tag{S1}$$

where

$$s^{(k)}(t)=E\left\{I(\widetilde X\geq t)\exp(\gamma^{\mathrm T}Z)Z^{\otimes k}\right\}\quad(k=0,1,2),$$
$$\mathcal E(t)=s^{(1)}(t)/s^{(0)}(t)$$
$$\text{and }\mathcal I=\int_0^\infty\left\{s^{(2)}(t)/s^{(0)}(t)-\mathcal E(t)^{\otimes 2}\right\}s^{(0)}(t)\mathrm d\Lambda_0(t).$$

On the other hand, the Breslow estimator is defined as

$$\hat\Lambda_0(t)=n^{-1}\sum_{i=1}^n\int_0^t\frac{\mathrm d\widetilde N_i(u)}{\sum_{j=1}^n I(\widetilde X_j\geq t)\exp(\widehat\gamma^{\mathrm T}Z_j)}.$$

Using a delta method on $\widehat{\gamma}$, we find that

$$n^{1/2}\{\widehat{\Lambda}_0(t)-\Lambda_0(t)\} = h(t)^{\mathrm{T}} n^{1/2}(\widehat{\gamma}-\gamma) + n^{-1/2}\sum_{i=1}^{n}\int_0^{\infty} s^{(0)}(u)^{-1}\mathrm{d}\widetilde{M}_i(u;\eta) + o_p(1), \tag{S2}$$

where

$$h(t) = -\int_0^t \mathcal{E}(u)\mathrm{d}\Lambda_0(u)$$

and we have used the fact that $E\{\mathrm{d}\widetilde{N}(u)\} = s^{(0)}(u)\mathrm{d}\Lambda(u)$.

Since $S(t \mid z;\eta) = \exp\left\{-\exp(\gamma^{\mathrm{T}}z)\Lambda_0(t)\right\}$, the derivative $\nabla S(t \mid z;\eta) := \partial S(t \mid z;\eta)/\partial\eta$ is given by

$$\begin{aligned}
&\nabla S(t \mid z;\eta)[\widehat{\gamma}-\gamma, \widehat{\Lambda}_0-\Lambda_0] \\
&= -S(t \mid z;\eta)\exp(\gamma^{\mathrm{T}}z)\left\{\Lambda_0(t)z^{\mathrm{T}}(\widehat{\gamma}-\gamma) + \widehat{\Lambda}_0(t)-\Lambda_0(t)\right\} \\
&= -S(t \mid z;\eta)\exp(\gamma^{\mathrm{T}}z)\left[\{\Lambda_0(t)z + H(t)\}^{\mathrm{T}}(\widehat{\gamma}-\gamma) + n^{-1}\sum_{i=1}^{n}\int_0^t s^{(0)}(u)\mathrm{d}\widetilde{M}_i(u;\eta)\right] + o_p(n^{-1/2}) \quad \text{(Plug in (S2))} \\
&= -n^{-1}\sum_{i=1}^{n} S(t \mid z;\eta)\left\{H(t;z)^{\mathrm{T}}\mathcal{I}^{-1}\int_0^{\infty}\{Z_i-\mathcal{E}(u)\}\mathrm{d}\widetilde{M}_i(u;\eta) + \exp(\gamma^{\mathrm{T}}z)\int_0^t s^{(0)}(u)^{-1}\mathrm{d}\widetilde{M}_i(u;\eta)\right\} + o_p(n^{-1/2}), \quad \text{(Plug in (S1))}
\end{aligned}$$

where

$$H(t;z) = \exp(\gamma^{\mathrm{T}}z)\int_0^t \{z-\mathcal{E}(u)\}\mathrm{d}\Lambda_0(u).$$

By the chain rule,

$$\begin{aligned}
\nabla\Omega_\eta(t;z,z^*)[\widehat{\gamma}-\gamma,\widehat{\Lambda}_0-\Lambda_0] &= -\mu(z,z^*;\beta)\left\{S(t \mid z^*;\eta)\nabla S(t \mid z;\eta) + S(t \mid z;\eta)\nabla S(t \mid z^*;\eta)\right\}[\widehat{\gamma}-\gamma,\widehat{\Lambda}_0-\Lambda_0] \\
&= n^{-1}\sum_{i=1}^{n}\mu(z,z^*;\beta)S(t \mid z;\eta)S(t \mid z^*;\eta)\Bigg[\{H(t;z)+H(t;z^*)\}\mathcal{I}^{-1}\int_0^{\infty}\left\{Z_i-\mathcal{E}(u)\right\}\mathrm{d}\widetilde{M}_i(u;\eta) \\
&\quad + \{\exp(\gamma^{\mathrm{T}}z)+\exp(\gamma^{\mathrm{T}}z^*)\}\int_0^t s^{(0)}(u)^{-1}\mathrm{d}\widetilde{M}_i(u;\eta)\Bigg] + o_p(n^{-1/2}).
\end{aligned}$$

The result follows by replacing the unknown quantities in the influence function with their estimators.

S1.2 | Asymptotic results under stratified PW and Cox models

For convenience, we redefine notation by using subscript (l) to represent a generic subject in the lth stratum ($l = 1,\ldots,L$) and subscript $(l)i$ to represent the ith subject ($i = 1,\ldots,n_l$), where n_l is the stratum size (with $\sum_{l=1}^{L} n_l = n$). Then the observed data consist of

$$\left\{\boldsymbol{Y}_{(l)i}\left(X_{(l)i}\right), X_{(l)i}, Z_{(l)i}\right\}, \quad i = 1,\ldots,n_l; l = 1,\ldots,L.$$

As the maximum *stratified* partial-likelihood estimator, $\widehat{\gamma}$ solves

$$n^{-1}\sum_{l=1}^{L}\sum_{i=1}^{n_l}\int_0^{\infty}\left\{Z_{(l)i} - \frac{\sum_{j=1}^{n_l} I(\widetilde{X}_{(l)j} \geq t)\exp(\widehat{\gamma}^{\mathrm{T}}Z_{(l)j})Z_j}{\sum_{j=1}^{n_l} I(\widetilde{X}_{(l)j} \geq t)\exp(\widehat{\gamma}^{\mathrm{T}}Z_{(l)j})}\right\}\mathrm{d}\widetilde{N}_{(l)i}(t) = 0.$$

S1.2.1 | Stratified Cox model estimators

Similarly to (S1), we find that

$$n^{1/2}(\widehat{\gamma}-\gamma) = n^{-1/2}\sum_{l=1}^{L}\sum_{i=1}^{n_l}\mathcal{I}^{-1}\int_0^{\infty}\left\{Z_{(l)i}-\mathcal{E}_l(t)\right\}\mathrm{d}\widetilde{M}_{(l)i}(t;\eta_l) + o_p(1), \tag{S3}$$

where

$$s_l^{(k)}(t) = E\left\{ I(\widetilde{X}_{(l)} \geq t)\exp(\gamma^{\mathrm{T}} Z_{(l)}) Z_{(l)}^{\otimes k} \right\} \quad (k = 0, 1, 2),$$

$$\mathcal{E}_l(t) = s_l^{(1)}(t)/s_l^{(0)}(t)$$

$$\mathcal{I}_l = \int_0^\infty \left\{ s_l^{(2)}(t)/s_l^{(0)}(t) - \mathcal{E}_l(t)^{\otimes 2} \right\} s^{(0)}(t)\mathrm{d}\Lambda_{0l}(t),$$

$$\mathcal{I} = \sum_{l=1}^L p_l \mathcal{I}_l, \text{ and } p_l = \lim_{n\to\infty} n_l/n.$$

Likewise for the Breslow estimator,

$$\hat{\Lambda}_{0l}(t) = n_l^{-1} \sum_{i=1}^{n_l} \int_0^t \frac{\mathrm{d}\widetilde{N}_{(l)i}(u)}{\sum_{j=1}^n I(\widetilde{X}_{(l)j} \geq t)\exp(\hat{\gamma}^{\mathrm{T}} Z_{(l)j})}.$$

Similarly to (S2), we find that

$$n^{1/2}\{\hat{\Lambda}_{0l}(t) - \Lambda_{0l}(t)\} = h_l(t)^{\mathrm{T}} n^{1/2}(\hat{\gamma} - \gamma) + n^{-1/2} p_l^{-1} \sum_{i=1}^{n_l} \int_0^\infty s_l^{(0)}(u)^{-1} \mathrm{d}\widetilde{M}_{(l)i}(u;\eta) + o_p(1), \tag{S4}$$

where $h_l(t) = -\int_0^t \mathcal{E}_l(u)\mathrm{d}\Lambda_{0l}(u)$.

Since $S(t \mid z; \eta_l) = \exp\left\{-\exp(\gamma^{\mathrm{T}} z)\Lambda_{0l}(t)\right\}$, the derivative $\nabla S(t \mid z; \eta_l) := \partial S(t \mid z; \eta_l)/\partial \eta_l$ is given by

$$\begin{aligned}
&\nabla S(t \mid z; \eta_l)[\hat{\gamma} - \gamma, \hat{\Lambda}_{0l} - \Lambda_{0l}] \\
&= -S(t \mid z; \eta_l)\exp(\gamma^{\mathrm{T}} z)\left\{ \Lambda_{0l}(t) z^{\mathrm{T}}(\hat{\gamma} - \gamma) + \hat{\Lambda}_{0l}(t) - \Lambda_{0l}(t) \right\} \\
&= -S(t \mid z; \eta_l)\exp(\gamma^{\mathrm{T}} z)\left[\{\Lambda_{0l}(t) z + H_l(t)\}^{\mathrm{T}}(\hat{\gamma} - \gamma) + n_l^{-1} \sum_{i=1}^{n_l} \int_0^t s_k^{(0)}(u)\mathrm{d}\widetilde{M}_{(l)i}(u;\eta_l) \right] + o_p(n^{-1/2}) \qquad \text{(Plug in (S4))} \\
&= -n^{-1} \sum_{l'=1}^L \sum_{i=1}^{n_{l'}} S(t \mid z; \eta_{l'}) \left\{ H_{l'}(t; z)^{\mathrm{T}} \mathcal{I}^{-1} \int_0^\infty \{Z_{(l')i} - \mathcal{E}_{l'}(u)\}\mathrm{d}\widetilde{M}_{(l')i}(u;\eta) + I(l' = l) p_l^{-1} \exp(\gamma^{\mathrm{T}} z) \int_0^t s_l^{(0)}(u)^{-1}\mathrm{d}\widetilde{M}_{(l)i}(u;\eta) \right\} \\
&\quad + o_p(n^{-1/2}), \qquad \text{(Plug in (S3))}
\end{aligned}$$

where

$$H_l(t; z) = \exp(\gamma^{\mathrm{T}} z) \int_0^t \{z - \mathcal{E}_l(u)\}\mathrm{d}\Lambda_{0l}(u).$$

By the chain rule,

$$\begin{aligned}
&\nabla \Omega_\eta(t; z, z^*)[\hat{\gamma} - \gamma, \hat{\Lambda}_0 - \Lambda_0] \\
&\equiv \frac{\partial}{\partial \eta_l} w_l(t \mid z, z^*)[\hat{\gamma} - \gamma, \hat{\Lambda}_0 - \Lambda_0] \\
&= -\mu(z, z^*; \beta)\left\{ S(t \mid z^*; \eta_l)\nabla S(t \mid z; \eta_l) + S(t \mid z; \eta_l)\nabla S(t \mid z^*; \eta_l) \right\}[\hat{\gamma} - \gamma, \hat{\Lambda}_0 - \Lambda_0] \\
&= n^{-1} \sum_{l'=1}^L \sum_{i=1}^{n_{l'}} \mu(z, z^*; \beta) S(t \mid z; \eta_{l'}) S(t \mid z^*; \eta_{l'}) \left[\left\{ H_{l'}(t; z) + H_{l'}(t; z^*) \right\} \mathcal{I}^{-1} \int_0^\infty \left\{ Z_{(l')i} - \mathcal{E}_{l'}(u) \right\} \mathrm{d}\widetilde{M}_{(l')i}(u;\eta) \right. \\
&\quad \left. + I(l' = l) p_l^{-1} \left\{ \exp(\gamma^{\mathrm{T}} z) + \exp(\gamma^{\mathrm{T}} z^*) \right\} \int_0^t s_l^{(0)}(u)^{-1}\mathrm{d}\widetilde{M}_{(l)i}(u;\eta) \right] + o_p(n^{-1/2}).
\end{aligned}$$

S1.2.2 | Stratified PW estimators

Use subscript $(l)ij$ to represent a pairwise quantity defined on subjects i and j in the lth stratum. Then Proposition 1 of Wang and Mao (2022)[1] shows that, under the standard weight $h_l(\cdot) = n_l$,

$$n^{1/2}(\hat{\beta} - \beta) = -2n^{1/2} \sum_{l=1}^{L} \sum_{i=1}^{n_l} A(\beta)^{-1} \kappa_l(\mathcal{O}_{(l)i}; \beta) + o_p(1),$$

where

$$A_l(\beta) = -E\left[R_{(l)ij}(\infty)\mu(Z_{(l)i}, Z_{(l)j}; \beta)\{1 - \mu(Z_{(l)i}, Z_{(l)j}; \beta)\}(Z_{(l)i} - Z_{(l)j})^{\otimes 2}\right],$$

$$A(\beta) = \sum_{l=1}^{L} p_l A_l(\beta),$$

$$\text{and } \kappa(\mathcal{O}_{(l)i}; \beta) = E\left\{(Z_{(l)i} - Z_{(l)j})M_{(l)ij}(\infty \mid Z_{(l)i}, Z_{(l)j}; \beta) \mid \mathcal{O}_{(l)i}\right\}.$$

By the chain rule,

$$\frac{\partial}{\partial \beta}\mu(z, z^*; \beta)[\hat{\beta} - \beta] = \mu(z, z^*; \beta)\{1 - \mu(z, z^*; \beta)\}(z - z^*)^{\mathrm{T}}(\hat{\beta} - \beta) + o_p(n^{-1/2})$$

$$= -2n^{1/2} \sum_{l=1}^{L} \sum_{i=1}^{n_l} \mu(z, z^*; \beta)\{1 - \mu(z, z^*; \beta)\}(z - z^*)^{\mathrm{T}} A(\beta)^{-1} \kappa_l(\mathcal{O}_{(l)i}; \beta) + o_p(n^{-1/2}).$$

S1.2.3 | Final results

Combining results from Sections S1.2.1 and S1.2.2, we obtain the asymptotic linear expansion of $\hat{w}(t \mid z, z^*)$.

Proposition S1. Under stratified PW and Cox models, given z and z^*, we have that

$$\begin{aligned} &n^{1/2}\{\hat{w}(t \mid z, z^*) - w(t \mid z, z^*)\} \\ =&n^{-1/2} \sum_{l'=1}^{L} \sum_{i=1}^{n_{l'}} \mu(z, z^*; \beta)\Bigg(S(t \mid z; \eta_{l'})S(t \mid z^*; \eta_{l'})\Big[\left\{H_{l'}(t; z) + H_{l'}(t; z^*)\right\} \mathcal{I}^{-1} \int_0^\infty \left\{Z_{(l')i} - \mathcal{E}_{l'}(u)\right\} \mathrm{d}\widetilde{M}_{(l')i}(u; \eta) \\ &+ I(l' = l)p_l^{-1} \left\{\exp(\gamma^{\mathrm{T}} z) + \exp(\gamma^{\mathrm{T}} z^*)\right\} \int_0^t s_l^{(0)}(u)^{-1} \mathrm{d}\widetilde{M}_{(l)i}(u; \eta)\Big] - 2\{1 - \mu(z, z^*; \beta)\}(z - z^*)^{\mathrm{T}} A(\beta)^{-1} \kappa_l(\mathcal{O}_{(l)i}; \beta)\Bigg) \\ &+ o_p(1). \end{aligned}$$

Replace the unknown quantities in the above with their estimators for to estimate the variance of $\hat{w}(t \mid z, z^*)$.

S1.3 | Some technical details for model (16)

Under model (16), since $v(t \mid z, z^*) + w(t \mid z, z^*) + w(t \mid z^*, z) = 1$, we can obtain

$$w(t \mid z, z^*) = \frac{g^{-1}\{\alpha(t) + \beta_w(t)^{\mathrm{T}} z + \beta_l(t)^{\mathrm{T}} z^*\}}{1 + g^{-1}\{\alpha(t) + \beta_w(t)^{\mathrm{T}} z + \beta_l(t)^{\mathrm{T}} z^*\} + g^{-1}\{\alpha(t) + \beta_w(t)^{\mathrm{T}} z^* + \beta_l(t)^{\mathrm{T}} z\}}, \tag{S5}$$

where $g^{-1}(\cdot)$ may be replaced by $\exp(\cdot)$ under a log link. This quantity can be evaluated by plugging in estimates of $\theta(t) := \{\alpha(t), \beta_w(t), \beta_l(t)\}$.

To estimate $\theta(t)$, recall that $w(t \mid Z_i, Z_j) = \mathrm{pr}\{\mathcal{W}(\boldsymbol{Y}_i, \boldsymbol{Y}_j)(t) = 1 \mid Z_i, Z_j\}$. However, the win indicator $\mathcal{W}(\boldsymbol{Y}_i, \boldsymbol{Y}_j)(t)$ is not always observable due to censoring. To address the censoring bias associated with $\delta_{ij}(t)$, we extend the inverse probability censoring weighting (IPCW) approach of Dong et al.[2] from the two-sample setting to the regression case. Specifically, note that $\mathcal{W}(\boldsymbol{Y}_i, \boldsymbol{Y}_j)(t)$ is observed definitively only if the censoring times C_i and C_j both exceed the "loser's" survival time (when win–loss is determined by death) or t (when determined by a lower-ranking component). This motivates the inversely weighted win indicator

$$\frac{I(C_i \wedge C_j \geq D_j \wedge t)}{G(X_j \wedge t \mid Z_i)\, G(X_j \wedge t \mid Z_j)} \mathcal{W}(\boldsymbol{Y}_i, \boldsymbol{Y}_j)(t),$$

where $G(t \mid Z) = \mathrm{pr}(C \geq t \mid Z)$, which we estimate using a Cox model to obtain $\hat{G}(t \mid Z)$.

By comparing all ordered pairs in the sample, we form the estimating function

$$V_n\{\theta(t)\} = \{n(n-1)\}^{-1} \sum_{i=1}^{n} \sum_{j \neq i} \begin{pmatrix} 1 \\ Z_i \\ Z_j \end{pmatrix} \left[\frac{I(C_i \wedge C_j \geq D_j \wedge t)}{\hat{G}(X_j \wedge t \mid Z_i)\hat{G}(X_j \wedge t \mid Z_j)} \mathcal{W}(\boldsymbol{Y}_i, \boldsymbol{Y}_j)(t) - w\{t \mid Z_i, Z_j; \theta(t)\} \right], \tag{S6}$$

where $w\{t \mid Z_i, Z_j; \theta(t)\}$ is the model-based win probability from (S5) with (z, z^*) replaced by (Z_i, Z_j). For each t, solving $V_n\{\hat{\theta}(t)\} = 0$ yields a $(2p+1)$-dimensional estimate $\hat{\theta}(t)$, with no constraints linking values across different t. Variance estimation must account for pairwise correlation (as a V-statistic) and the additional randomness in $\hat{G}(t \mid Z)$.

Remark 1. This framework is applicable when a quantitative biomarker is a component of the hierarchical endpoint, provided two conditions hold. First, $\mathcal{W}(\boldsymbol{Y}_i, \boldsymbol{Y}_j)(t)$ depends on biomarker values collected during $[0, t]$; second, there is no missing data in this interval. If the biomarker has intermittent missingness even while the subject remains under observation, additional weighting is required for unbiased estimation.

S2 | ADDITIONAL NUMERICAL RESULTS

S2.1 | Additional simulation results

To validate the model fitting procedures, the estimation and inference of the regression coefficients are collected and summarized in Table S1.

TABLE S1 Estimation and inference of β under the PW model.

n	Term	Bias	SE	SEE	CP
100	$Z_{\cdot 1}$	0.019	0.146	0.145	0.948
	$Z_{\cdot 2}$	−0.001	0.135	0.134	0.953
	$Z_{\cdot 3}$	−0.017	0.272	0.272	0.952
200	$Z_{\cdot 1}$	0.010	0.103	0.100	0.938
	$Z_{\cdot 2}$	−0.002	0.094	0.092	0.947
	$Z_{\cdot 3}$	−0.005	0.182	0.187	0.958
500	$Z_{\cdot 1}$	0.006	0.064	0.063	0.945
	$Z_{\cdot 2}$	0.001	0.060	0.058	0.940
	$Z_{\cdot 3}$	−0.003	0.121	0.117	0.943
1000	$Z_{\cdot 1}$	0.004	0.044	0.044	0.950
	$Z_{\cdot 2}$	−0.001	0.040	0.041	0.948
	$Z_{\cdot 3}$	−0.001	0.083	0.082	0.952
2000	$Z_{\cdot 1}$	0.002	0.032	0.031	0.947
	$Z_{\cdot 2}$	0.001	0.029	0.029	0.951
	$Z_{\cdot 3}$	-0.003	0.058	0.058	0.954

SE: Empirical standard deviation of estimator;
SEE: Empirical mean of standard error estimator;
CP: Empirical coverage probability of 95% confidence interval;
Each scenario is based on 2,000 replicates.

Corresponding to Table 1 of the main text, inferential results for the loss probabilities are summarized in Table S2.

TABLE S2 Estimation and inference of loss probabilities at specific t's.

		$w(t \mid z_2, z_1)$					$w(t \mid z_3, z_2)$				
n	t	True	Bias	SE	SEE	CP	True	Bias	SE	SEE	CP
100	0.05	0.029	−0.001	0.013	0.013	0.956	0.047	−0.002	0.018	0.018	0.961
	0.10	0.056	−0.001	0.019	0.019	0.950	0.088	−0.003	0.025	0.026	0.961
	1.00	0.302	−0.005	0.043	0.043	0.948	0.351	−0.006	0.033	0.033	0.947
	4.00	0.377	−0.007	0.034	0.034	0.949	0.378	−0.004	0.034	0.034	0.948
200	0.05	0.029	0.000	0.009	0.009	0.943	0.047	−0.001	0.013	0.013	0.950
	0.10	0.056	0.000	0.014	0.014	0.948	0.088	−0.001	0.018	0.018	0.951
	1.00	0.302	−0.003	0.032	0.030	0.936	0.351	−0.003	0.023	0.023	0.935
	4.00	0.377	−0.004	0.024	0.024	0.936	0.378	−0.002	0.024	0.023	0.937
500	0.05	0.029	0.000	0.006	0.006	0.965	0.047	0.000	0.008	0.008	0.960
	0.10	0.056	0.000	0.009	0.009	0.945	0.088	−0.001	0.011	0.012	0.951
	1.00	0.302	−0.002	0.020	0.019	0.940	0.351	−0.002	0.015	0.014	0.940
	4.00	0.377	−0.002	0.015	0.015	0.943	0.378	−0.001	0.015	0.015	0.945
1000	0.05	0.029	0.000	0.004	0.004	0.948	0.047	0.000	0.006	0.006	0.949
	0.10	0.056	0.000	0.006	0.006	0.951	0.088	0.000	0.008	0.008	0.950
	1.00	0.302	−0.001	0.013	0.013	0.950	0.351	−0.001	0.010	0.010	0.948
	4.00	0.377	−0.001	0.010	0.010	0.948	0.378	−0.001	0.010	0.010	0.950

See note to Table S1.

S2.2 | Additional analysis of the real example

Cox model results for the TFE are summarized in Table S3.

TABLE S3 Cox model results for time to first event in the HF-ACTION study.

	HR	95% CI	p-value
Training vs UC	0.90	[0.81, 1.02]	0.082
Age (years)	1.00	[0.99, 1.00]	0.784
Female vs Male	0.81	[0.71, 0.92]	<0.001
Non-White vs White	1.17	[1.04, 1.31]	0.009
Ischemic vs Non-ischemic	1.11	[0.99, 1.25]	0.081
BMI	0.99	[0.98, 1.00]	0.038
CPX duration (min)	1.20	[1.07, 1.35]	0.003
NYHA class III vs II	1.82	[1.15, 2.9]	0.011
NYHA class IV vs II	0.93	[0.91, 0.95]	<0.001
Six-minute walk distance (m)	0.93	[0.87, 0.99]	0.020
Best LVEF (%)	0.98	[0.97, 0.99]	<0.001

Martingale residual analysis for the Cox TFE model is plotted in Figure S1. Of note, CPX duration shows an upward curve beyond 20 minutes, due to an excess of events compared to model predictions based on a linear effect.

Numerical results from Figure 3 of the main text are tabulated for years 1, 2, 3, and 4 in Table S4.

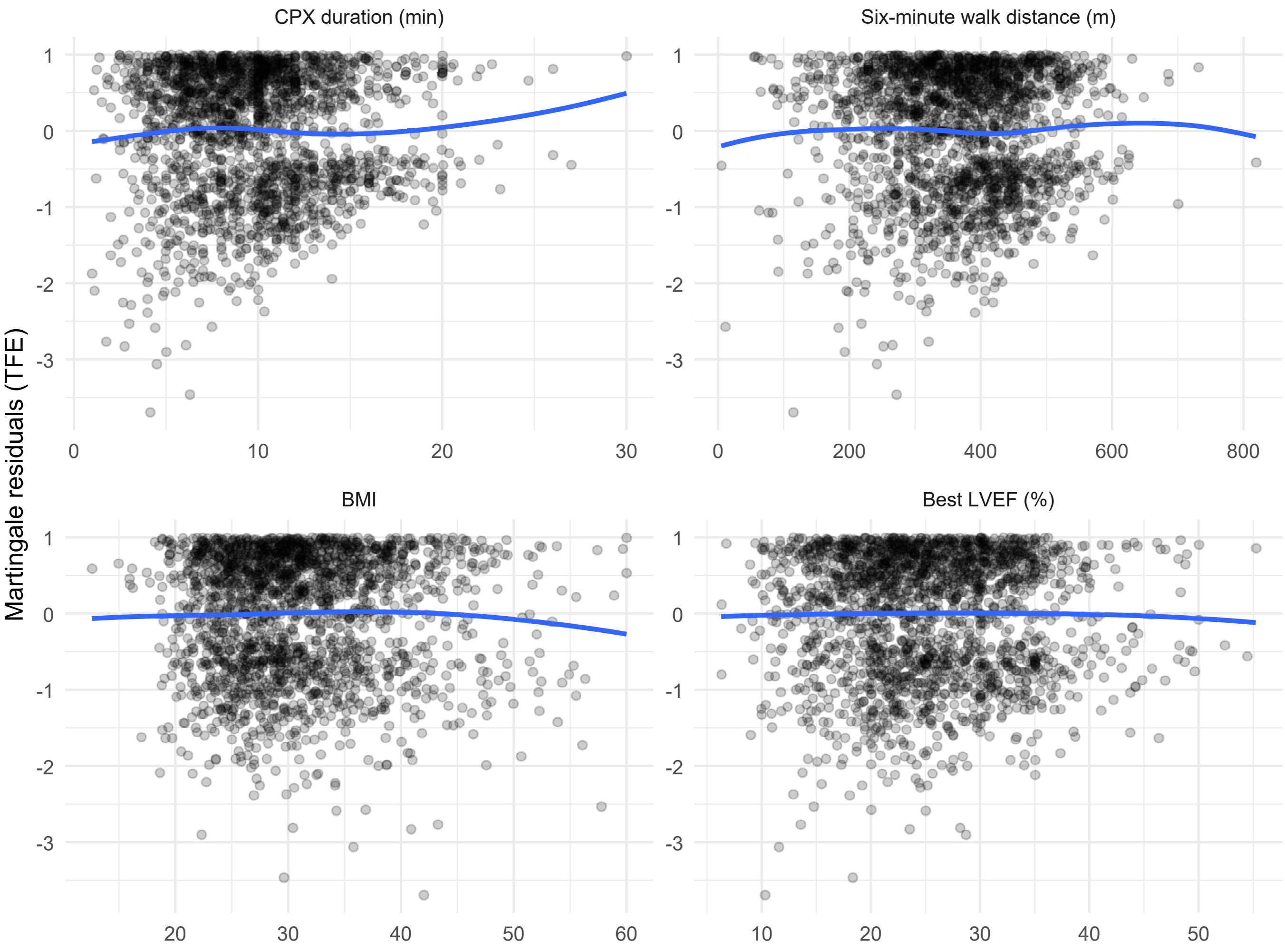

FIGURE S1 Residual analysis for initial Cox model for the TFE also shows nonlinear effect of CPX duration beyond 20 mins.

To further assess the linearity of CPX, we refit the PW model using grouped CPX values at approximately 4–5 minute intervals. The resulting log-win ratio estimates relative to the baseline group are shown in Figure S2. The estimates exhibit an approximately linear trend across categories, supporting the use of a continuous specification for CPX in the final model.

Under this model with discrete CPX values, the predicted win probabilities comparing different groups are overlaid with those obtained under the continuous specification in Figure S3. The close agreement between the two sets of estimates further supports the adequacy of the linearity assumption for CPX.

References

1. Wang T, Mao L. Stratified proportional win-fractions regression analysis. *Statistics in Medicine* 2022; 41(26): 5305–5318.

2. Dong G, Mao L, Huang B, et al. The inverse-probability-of-censoring weighting (IPCW) adjusted win ratio statistic: an unbiased estimator in the presence of independent censoring. *Journal of Biopharmaceutical Statistics* 2020; 30(5): 882–899. doi: 10.1080/10543406.2020.1778949

TABLE S4 Predicted win-loss probabilities and contrast measures comparing CPX durations in the HF-ACTION study.

Year	Pair	Win/Loss	Win odds	Net benefit
1	CPX: 5 mins vs 1 min	0.49/0.35	1.32 [1.22, 1.43]	0.14 [0.10, 0.18]
	CPX: 10 mins vs 5 mins	0.44/0.29	1.35 [1.25, 1.47]	0.15 [0.11, 0.19]
	CPX: 15 mins vs 10 mins	0.36/0.24	1.28 [1.21, 1.36]	0.12 [0.10, 0.15]
	CPX: 20+ mins vs 15 mins	0.29/0.19	1.22 [1.17, 1.27]	0.10 [0.08, 0.12]
2	CPX: 5 mins vs 1 min	0.56/0.40	1.38 [1.26, 1.50]	0.16 [0.12, 0.20]
	CPX: 10 mins vs 5 mins	0.55/0.36	1.46 [1.32, 1.61]	0.19 [0.14, 0.23]
	CPX: 15 mins vs 10 mins	0.49/0.32	1.40 [1.29, 1.51]	0.17 [0.13, 0.20]
	CPX: 20+ mins vs 15 mins	0.41/0.27	1.33 [1.26, 1.40]	0.14 [0.11, 0.17]
3	CPX: 5 mins vs 1 min	0.58/0.41	1.39 [1.28, 1.51]	0.16 [0.12, 0.20]
	CPX: 10 mins vs 5 mins	0.58/0.38	1.49 [1.34, 1.65]	0.20 [0.15, 0.25]
	CPX: 15 mins vs 10 mins	0.54/0.36	1.45 [1.33, 1.59]	0.18 [0.14, 0.23]
	CPX: 20+ mins vs 15 mins	0.48/0.32	1.39 [1.30, 1.49]	0.16 [0.13, 0.20]
4	CPX: 5 mins vs 1 min	0.58/0.42	1.39 [1.28, 1.52]	0.16 [0.12, 0.21]
	CPX: 10 mins vs 5 mins	0.59/0.39	1.51 [1.36, 1.67]	0.20 [0.15, 0.25]
	CPX: 15 mins vs 10 mins	0.57/0.38	1.48 [1.35, 1.63]	0.19 [0.15, 0.24]
	CPX: 20+ mins vs 15 mins	0.53/0.35	1.44 [1.33, 1.56]	0.18 [0.14, 0.22]

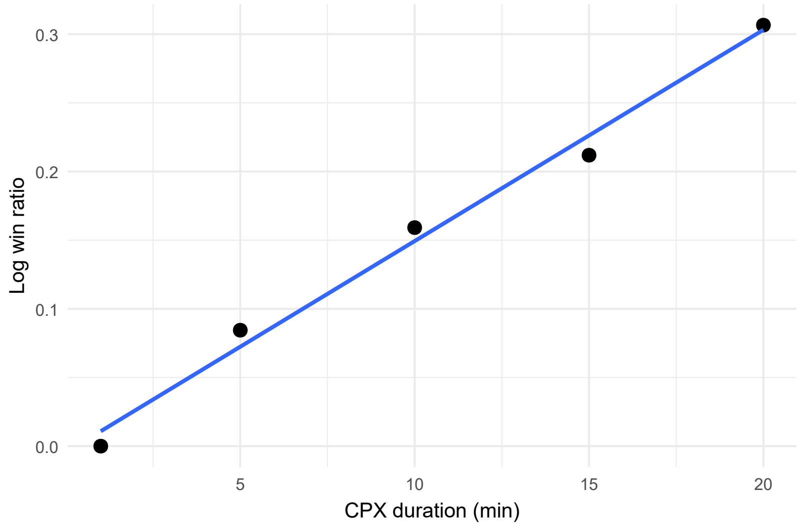

FIGURE S2 Estimated log-win ratios across categorized CPX duration (relative to the baseline group), showing an approximately linear trend.

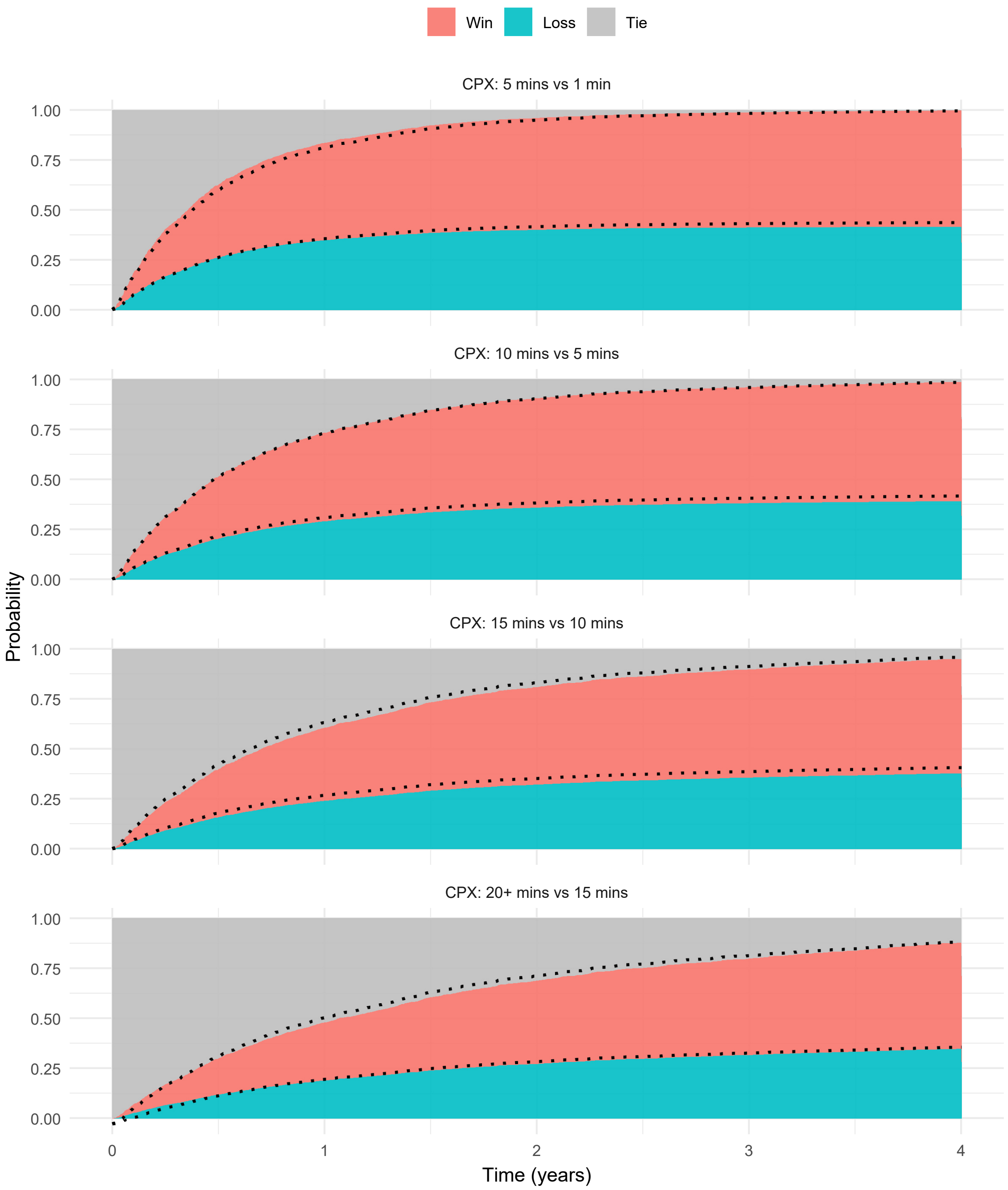

FIGURE S3 Predicted win probabilities comparing CPX groups based on the categorized (dotted lines) and continuous (areas) specifications, showing close agreement between the two approaches.
