# Supplementary material for: Predicting Win‐Loss Probabilities for Composite Time‐to‐Event Outcomes Under The Proportional Win‐Fractions Regression Model
Source: Stat Med. 2026 Apr 28;45:e70569. doi: 10.1002/sim.70569 (PMC13124461; doi:10.1002/sim.70569)
Supplement: Supplementary file 2 — Data S2: sim70569‐sup‐0002‐Supinfo.zip. [file SIM-45-0-s001.zip › R-code to reproduce results/wl_probs.html]

Numerical studies for prediction of win-loss probabilities under PW model


## Table of contents

- Basic functions
- Simulation studies
  - Data generating process
  - Table S1: Estimation and inference of \(\beta\_1\)
  - Figure 1. Estimation of win-loss curves
  - Example with misspecified models
  - Table 1: Inference of \(w(t\mid z, z^\*)\) at specific \(t\)’s
  - Table S2: Inference of \(w(t\mid z^\*, z)\) at specific \(t\)’s
  - Table 2: Inference on win odds and net benefits
  - Reproducible code example
- Real example
  - Table 3: Summary of the HF-ACTION dataset
  - Inital PW model
  - Figure 2 and Table 4
  - Table S3: Cox model results
  - Figure 3: Predicted win-loss probabilities, win ratio, win odds, and net benefit
  - Table S4: Predictions based on final model

# Numerical studies for prediction of win-loss probabilities under PW model

Author

Lu Mao

Published

December 2, 2025

\[
\newcommand{\indep}{\perp \!\!\! \perp}
\def\T{{ \mathrm{\scriptscriptstyle T} }}
\def\pr{{\rm pr}}
\def\d{{\rm d}}
\def\W{{\mathcal W}}
\def\H{{\mathcal H}}
\def\I{{\mathcal I}}
\def\E{{\mathcal E}}
\def\S{{\mathcal S}}
\def\O{{\mathcal O}}
\def\Un{{n\choose 2}^{-1}\sum\_{i<j}^n\sum}
\def\l{(l)}
\def\n{{(n)}}
\def\v{\varepsilon}
\def\bSig\mathbf{\Sigma}
\def\Gn{n^{-1/2}\sum\_{i=1}^n}
\def\Pni{(n-1)^{-1}\sum\_{j=1}^{n-1}}
\]

```
library(WR)
library(gumbel)
library(tidyverse)
library(knitr)
library(patchwork)
library(survival)
```

This document provides a step-by-step guide to repoduce the numerical results in the simulations studies (Section 3) and real data analysis (Section 4) in the manuscript.

## Basic functions

Code for basic functions

```
# Helper function of generate_gb_data ----------------------------
time_fun <- function(death_time, nonfatal_time, censor_time) {

  X <- min(death_time, censor_time)
  delta <- (death_time <= censor_time) + 0
  if (nonfatal_time < X) {
    data <- tibble(
      time = c(nonfatal_time, X),
      status = c(2, delta)
    )
  } else {
    data <- tibble(
      time = X,
      status = delta
    )
  }


  return(data)
}

# Simulate bivariate Gumbel random variables --------------------
generate_gb_data <- function(n, beta0, lambdaH, lambdaD, kappa, lambdaC, fmin, fmax,
                             f_Z1 = NULL) {
  # Censoring
  # Expn(lambdaC) \wedge U(fmin, fmax)
  C <- pmin(rexp(n, lambdaC), (fmax - fmin) * runif(n) + fmin)
  # Simulate bivariate Gumbel random variables
  outcomes <- rgumbel(n, alpha = kappa, dim = 2, method = 1)
  # Simulate covariates (Z1 Bernoulli(0.5); Z2 Z3: N(0, 1))
  Z <- tibble(
    Z1 = rnorm(n, 0, 1),
    # Z1 = 4 * runif(n),
    Z2 = rnorm(n, -1, 1),
    Z3 = rbinom(n, 1, 0.5)
  )
  # This only goes to outcome generation
  if (!is.null(f_Z1)) {
    Z_outcomes <- Z |>
      mutate(
        Z1 = f_Z1(Z1)
      )
  } else{
    Z_outcomes <- Z
  }


  Zmat <- as.matrix(Z_outcomes)

  # Generate outcomes based on conditional dist
  U1 <- outcomes[, 1]
  U2 <- outcomes[, 2]
  nonfatal_time <- -log(U1) / (lambdaH * exp(- Zmat %*% beta0))
  death_time <- -log(U2) / (lambdaD * exp(- Zmat %*% beta0))
  # Combine data to wide format
  df_wide <- tibble(
    id = 1:n,
    nonfatal_time = as.vector(nonfatal_time),
    death_time = as.vector(death_time),
    censor_time = C,
    Z
  )

  # Wide to long
  df <- df_wide |>
    group_by(id) |>
    reframe(
      ts = time_fun(death_time, nonfatal_time, censor_time)
    ) |>
    unnest(ts) |>
    left_join(
      tibble(id = 1:n, Z),
      by = "id")

  df
}


# Simulate bivariate Gumbel random variables --------------------
# Modified to have betaH and betaD separately, potentially misspecifying PW/PH models
generate_gb_data_misp <- function(n, betaH, betaD, lambdaH, lambdaD, kappa, lambdaC, fmin, fmax,
                             f_Z1 = NULL) {
  # Censoring
  # Expn(lambdaC) \wedge U(fmin, fmax)
  C <- pmin(rexp(n, lambdaC), (fmax - fmin) * runif(n) + fmin)
  # Simulate bivariate Gumbel random variables
  outcomes <- rgumbel(n, alpha = kappa, dim = 2, method = 1)
  # Simulate covariates (Z1 Bernoulli(0.5); Z2 Z3: N(0, 1))
  Z <- tibble(
    Z1 = rnorm(n, 0, 1),
    # Z1 = 4 * runif(n),
    Z2 = rnorm(n, -1, 1),
    Z3 = rbinom(n, 1, 0.5)
  )
  # This only goes to outcome generation
  if (!is.null(f_Z1)) {
    Z_outcomes <- Z |>
      mutate(
        Z1 = f_Z1(Z1)
      )
  } else{
    Z_outcomes <- Z
  }


  Zmat <- as.matrix(Z_outcomes)

  # Generate outcomes based on conditional dist
  U1 <- outcomes[, 1]
  U2 <- outcomes[, 2]
  nonfatal_time <- -log(U1) / (lambdaH * exp(- Zmat %*% betaH))
  death_time <- -log(U2) / (lambdaD * exp(- Zmat %*% betaD))
  # Combine data to wide format
  df_wide <- tibble(
    id = 1:n,
    nonfatal_time = as.vector(nonfatal_time),
    death_time = as.vector(death_time),
    censor_time = C,
    Z
  )

  # Wide to long
  df <- df_wide |>
    group_by(id) |>
    reframe(
      ts = time_fun(death_time, nonfatal_time, censor_time)
    ) |>
    unnest(ts) |>
    left_join(
      tibble(id = 1:n, Z),
      by = "id")

  df
}


# Model fitting  -----------------------------
fit_pw_model <- function(df) {

  id <- df$id
  time <- df$time
  status <- df$status
  Z <- df[, 4:ncol(df)]

  obj <- pwreg1(id, time, status, Z)

  return(obj)

}


# Extract results from fitted models --------------------------------------

## function to extract results from a single model
extract_beta <- function(obj) {
  list(
    estimate = obj$beta,
    std.error = sqrt(diag(obj$Var))
  )
}

## Extract results from all models
tidy_beta_est_err <- function(simulation_results, by_n = FALSE) {

  stats <- simulation_results |>
    mutate(
      statistics = map(obj, extract_beta)
    ) |>
    unnest_wider(statistics) |>
    unnest_longer(c(estimate, std.error))


  if (by_n) {
    stats |>
    select(n, sim_id,
           term = estimate_id,
           estimate,
           std.error)
    } else {
    stats |>
      select(sim_id,
           term = estimate_id,
           estimate,
           std.error)
  }
}


# Summarize results across simulated datasets -----------------------------

summarize_beta <- function(stats, beta0, alpha = 0.05) {

  za <- qnorm(1 - alpha / 2)

  terms <- unique(stats$term)

  beta0_tibble <- tibble(
    term = terms,
    beta0 = beta0
  )

  stats |>
    left_join(beta0_tibble) |>
    group_by(term) |>
    summarize(
      bias = mean(estimate - beta0),
      SE = sd(estimate),
      SEE = mean(std.error),
      coverage = mean((beta0 > estimate - za * std.error) & (beta0 < estimate + za * std.error))
    )
}


summarize_beta_by_n <- function(stats, beta0, alpha = 0.05) {

  za <- qnorm(1 - alpha / 2)

  terms <- unique(stats$term)

  beta0_tibble <- tibble(
    term = terms,
    beta0 = beta0
  )

  stats |>
    left_join(beta0_tibble) |>
    group_by(n, term) |>
    summarize(
      bias = mean(estimate - beta0),
      SE = sd(estimate),
      SEE = mean(std.error),
      coverage = mean((beta0 > estimate - za * std.error) & (beta0 < estimate + za * std.error))
    )
}


# Extract prediction results from fitted models ----------------------------

## function to extract results from a single model's results
extract_preds <- function(preds, t) {

  preds_t <- tibble(
    t = t,
    results = map(t, \(x) preds |> filter(time <= x)
                        |> slice_tail(n = 1))
      )

    # unnest_wider(results)
  preds_t
}


# Transformed confidence intervals ----------------------------------------

logit <- function(x) log(x / (1 - x))
expit <- function(x) exp(x) / (1 + exp(x))
logit_prm <- function(x) ifelse(x == 0 | x == 1, 0, (x * (1 - x))^{-1})


logit_ci <- function(prop, se, alpha = 0.05) {
  za <- qnorm(1 - alpha / 2)
  prop_logit <- logit(prop)
  se_logit <- logit_prm(prop) * se
  lower <- expit(prop_logit - za * se_logit)
  upper <- expit(prop_logit + za * se_logit)
  list(lower = lower, upper = upper)
}
```

## Simulation studies

### Data generating process

- Covariates: \(Z = (Z\_1, Z\_2, Z\_3)^{\rm T}\) with mutually independent components
  - \(Z\_1\sim N(0, 1)\)
  - \(Z\_2\sim N(-1, 1)\)
  - \(Z\_3\sim \mbox{Bernoulli}(0.5)\)
- Outcome model: \[\begin{equation}\tag{1}
  \pr(D > s, T > t\mid Z) =
  \exp\left(-\left[\{\exp(-\beta^\T Z)\lambda\_Ds\}^\kappa + \{\exp(-\beta^\T Z)\lambda\_Ht\}^\kappa\right]^{1/\kappa}\right)
  \end{equation}\]
  - \(\lambda\_D = 0.1\)
  - \(\lambda\_H = 1\)
  - \(\kappa = 2\)
  - \(\beta\_0 = (0.5, 0, -0.5)^\T\)
- Censoring: \(\mbox{Un}[1, 4]\wedge\mbox{Expn}(\lambda\_C)\)
  - \(\lambda\_C = 0.02\)

Model parameter specifications

```
## Individual experiments
# Fix parameters
kappa <- 2
#hazard rates
lambdaH <- 1
lambdaD <- 0.1
# lambdaD <- 1
#censoring
lambdaC <- 0.02
fmin <- 0.2
fmax <- 4
# beta0=c(0,-0)
beta0 <- c(0.5, 0, -0.5)
```

Generate data

```
library(WR)
library(gumbel)
library(tidyverse)

source("basic_functions.R")
source("model_parameter_specification.R")

N <- 2000
set.seed(123)
## Function to simulate N datasets with n samples each

simulate_n <- function(N, n, beta0, lambdaH, lambdaD, kappa, lambdaC, fmin, fmax, f_Z1 = NULL){
  tibble(
    sim_id = 1:N
  ) %>%
    mutate(
      data = map(sim_id, ~ generate_gb_data(n, beta0, lambdaH, lambdaD, kappa, lambdaC, fmin, fmax,
                                            f_Z1 = f_Z1)) # Generate datasets
    )
}


# Generate data with different sample size n
n_list <- c(100, 200, 500, 1000, 2000)

for (n in n_list){
  data = simulate_n(N, n, beta0, lambdaH, lambdaD, kappa, lambdaC, fmin, fmax)
  saveRDS(data, paste0("simulated_datasets/data_n", n, ".rds"))
  rm(data)
}
```

### Table S1: Estimation and inference of \(\beta\_1\)

Model fitting code

```
library(WR)
library(gumbel)
library(tidyverse)

source("basic_functions.R")

# Model fitting for different sample size n --------------------------------
n_list <- c(100, 200, 500, 1000)

for (n in n_list){
  mod_fit <- readRDS(paste0("simulated_datasets/data_n", n, ".rds")) |>
    mutate(
    obj = map(data, fit_pw_model)
  )
saveRDS(mod_fit, paste0("model_fitting_results/mod_fit_n", n, ".rds"))
rm(mod_fit)
}


# Fit model for n = 2000 piecemeal to save memory ------------------------
n <- 2000

dat_n2000 <- readRDS(paste0("simulated_datasets/data_n", n, ".rds"))

mod_fit_n2000_p1 <- dat_n2000 |>
  filter(sim_id %in% c(1:500)) |>
  mutate(
    obj = map(data, fit_pw_model)
  )
saveRDS(mod_fit_n2000_p1, paste0("model_fitting_results/mod_fit_n", n, "p1.rds"))
rm(mod_fit_n2000_p1)

mod_fit_n2000_p2 <- dat_n2000 |>
  filter(sim_id %in% c(501:1000)) |>
  mutate(
    obj = map(data, fit_pw_model)
  )
saveRDS(mod_fit_n2000_p2, paste0("model_fitting_results/mod_fit_n", n, "p2.rds"))
rm(mod_fit_n2000_p2)

mod_fit_n2000_p3 <- dat_n2000 |>
  filter(sim_id %in% c(1001:1500)) |>
  mutate(
    obj = map(data, fit_pw_model)
  )
saveRDS(mod_fit_n2000_p3, paste0("model_fitting_results/mod_fit_n", n, "p3.rds"))
rm(mod_fit_n2000_p3)

mod_fit_n2000_p4 <- dat_n2000 |>
  filter(sim_id %in% c(1501:2000)) |>
  mutate(
    obj = map(data, fit_pw_model)
  )
saveRDS(mod_fit_n2000_p4, paste0("model_fitting_results/mod_fit_n", n, "p4.rds"))
rm(mod_fit_n2000_p4)
```

Summarize model-fitting results

```
source("basic_functions.R")
source("model_parameter_specification.R")


mod_fit_n100 <- readRDS(paste0("model_fitting_results/mod_fit_n", 100, ".rds"))
mod_fit_n200 <- readRDS(paste0("model_fitting_results/mod_fit_n", 200, ".rds"))
mod_fit_n500 <- readRDS(paste0("model_fitting_results/mod_fit_n", 500, ".rds"))
mod_fit_n1000 <- readRDS(paste0("model_fitting_results/mod_fit_n", 1000, ".rds"))

# tidy model results
mod_est_tidy_n100 <- tidy_beta_est_err(mod_fit_n100, by_n = FALSE)
mod_est_tidy_n200 <- tidy_beta_est_err(mod_fit_n200, by_n = FALSE)
mod_est_tidy_n500 <- tidy_beta_est_err(mod_fit_n500, by_n = FALSE)
mod_est_tidy_n1000 <- tidy_beta_est_err(mod_fit_n1000, by_n = FALSE)

# combine the tidied results
mod_est_tidy <- bind_rows(
  mod_est_tidy_n100 |> mutate(n = 100),
  mod_est_tidy_n200 |> mutate(n = 200),
  mod_est_tidy_n500 |> mutate(n = 500),
  mod_est_tidy_n1000 |> mutate(n = 1000)
)

## add n = 2000
mod_fit_n2000p1 <- readRDS(paste0("model_fitting_results/mod_fit_n", 2000, "p1.rds"))
mod_est_tidy_n2000p1 <- tidy_beta_est_err(mod_fit_n2000p1, by_n = FALSE)
rm(mod_fit_n2000p1)
mod_fit_n2000p2 <- readRDS(paste0("model_fitting_results/mod_fit_n", 2000, "p2.rds"))
mod_est_tidy_n2000p2 <- tidy_beta_est_err(mod_fit_n2000p2, by_n = FALSE)
rm(mod_fit_n2000p2)
mod_fit_n2000p3 <- readRDS(paste0("model_fitting_results/mod_fit_n", 2000, "p3.rds"))
mod_est_tidy_n2000p3 <- tidy_beta_est_err(mod_fit_n2000p3, by_n = FALSE)
rm(mod_fit_n2000p3)
mod_fit_n2000p4 <- readRDS(paste0("model_fitting_results/mod_fit_n", 2000, "p4.rds"))
mod_est_tidy_n2000p4 <- tidy_beta_est_err(mod_fit_n2000p4, by_n = FALSE)
rm(mod_fit_n2000p4)

# Combine
mod_est_tidy <- mod_est_tidy |> 
  bind_rows(
    mod_est_tidy_n2000p1 |> mutate(n = 2000),
    mod_est_tidy_n2000p2 |> mutate(n = 2000),
    mod_est_tidy_n2000p3 |> mutate(n = 2000),
    mod_est_tidy_n2000p4 |> mutate(n = 2000)
    
  )

# Summarize results for each n
tbl_results <- summarize_beta_by_n(mod_est_tidy, beta0) |> 
  mutate(
    n = if_else(row_number() == 1, as.character(n), "")
  ) |> 
  mutate(
    across(bias:coverage, ~ round(., 3))
  ) |> 
  rename(
    `Bias` = bias,
    `Term` = term,
    `Coverage` = coverage
  )

# Output in latex code
latex_code <- kable(tbl_results, format = "latex", booktabs = TRUE)

# Print LaTeX code
cat(latex_code)

kable(tbl_results)
# |n    |Term |   Bias|    SE|   SEE| Coverage|
# |:----|:----|------:|-----:|-----:|--------:|
# |100  |Z1   |  0.019| 0.146| 0.145|    0.948|
# |     |Z2   | -0.001| 0.135| 0.134|    0.953|
# |     |Z3   | -0.017| 0.272| 0.272|    0.952|
# |200  |Z1   |  0.010| 0.103| 0.100|    0.938|
# |     |Z2   | -0.002| 0.094| 0.092|    0.947|
# |     |Z3   | -0.005| 0.182| 0.187|    0.958|
# |500  |Z1   |  0.006| 0.064| 0.063|    0.945|
# |     |Z2   |  0.001| 0.060| 0.058|    0.940|
# |     |Z3   | -0.003| 0.121| 0.117|    0.943|
# |1000 |Z1   |  0.004| 0.044| 0.044|    0.950|
# |     |Z2   | -0.001| 0.040| 0.041|    0.948|
# |     |Z3   | -0.001| 0.083| 0.082|    0.952|
# |2000 |Z1   |  0.002| 0.032| 0.031|    0.947|
# |     |Z2   |  0.001| 0.029| 0.029|    0.951|
# |     |Z3   | -0.003| 0.058| 0.058|    0.954|
```

### Figure 1. Estimation of win-loss curves

Generate predictions based on fitted models

```
library(WR)
library(gumbel)
library(tidyverse)

source("basic_functions.R")


# Model fitting for different sample size n --------------------------------

z1 <- c(1, 0, 0)
z2 <- c(0, 0, 0)
z3 <- c(-1, 0, 0)


# Cut-off time points (ensure a smooth curve)
ts <- c(seq(0.05, 1, by = 0.05), seq(1.1, 2, by = 0.1), 2.2, 2.5, 3, 3.5, 4)

n_list <- c(100, 200, 500, 1000, 2000)

# n <- 100

for (n in n_list){

  mods <- readRDS(paste0("model_fitting_results/mod_fit_n", n, ".rds"))

  # z1 vs z2
  preds12 <- mods |>
    mutate(preds = map(obj, ~ predict(., z1, z2, contrast = TRUE)))
  preds12_t <- preds12 |>
    mutate(preds_t = map(preds, ~ extract_preds(., ts))) |>
    select(sim_id, preds_t) |>
    unnest(preds_t) |>
    unnest(results)

  saveRDS(preds12_t, paste0("model_predicting_results/preds12_t_n", n, ".rds"))
  rm(preds12, preds12_t)

  # z2 vs z3
  preds23 <- mods |>
    mutate(preds = map(obj, ~ predict(., z2, z3, contrast = TRUE)))
  preds23_t <- preds23 |>
    mutate(preds_t = map(preds, ~ extract_preds(., ts))) |>
    select(sim_id, preds_t) |>
    unnest(preds_t) |>
    unnest(results)

  saveRDS(preds23_t, paste0("model_predicting_results/preds23_t_n", n, ".rds"))
  rm(preds23, preds23_t)
}
```

Collect prediction results

```
source("basic_functions.R")
source("model_parameter_specification.R")

z1 <- c(1, 0, 0)
z2 <- c(0, 0, 0)
z3 <- c(-1, 0, 0)


# Calculate the true values ------------------------------
lambda <- (lambdaH^kappa + lambdaD^kappa)^{1/kappa}
# Cut-off time points (ensure a smooth curve)
times <- c(seq(0.05, 1, by = 0.05), seq(1.1, 2, by = 0.1), 2.2, 2.5, 3, 3.5, 4)
# True parameters for the Cox TFE model
gamma0 <- - beta0
Lambda0 <- lambda * times
# Survival functions for the TFE
Sz1t <- exp(- exp(sum(gamma0 * z1)) * Lambda0)
Sz2t <- exp(- exp(sum(gamma0 * z2)) * Lambda0)
Sz3t <- exp(- exp(sum(gamma0 * z3)) * Lambda0)
# Mu functions from PW model
mu12 <- exp(sum(beta0 * z1)) / (exp(sum(beta0 * z1)) + exp(sum(beta0 * z2)))
mu23 <- exp(sum(beta0 * z2)) / (exp(sum(beta0 * z2)) + exp(sum(beta0 * z3))) 
# Comparability (win or loss) probabilities
comp_probs12 <- 1 - Sz1t * Sz2t
comp_probs23 <- 1 - Sz2t * Sz3t
# Win and loss probabilities
win_prob012 <- comp_probs12 * mu12
loss_prob012 <- comp_probs12 - win_prob012
win_prob023 <- comp_probs23 * mu23
loss_prob023 <- comp_probs23 - win_prob023
# Tibble data frames for true values to be merged with simulated ones
true_values12 <- tibble(t = times, win_prob0 = win_prob012, loss_prob0 = loss_prob012)
true_values23 <- tibble(t = times, win_prob0 = win_prob023, loss_prob0 = loss_prob023)


# Read in the prediction results 
preds_stats <- tibble(NULL)

for (n in c(100, 200, 500, 1000)){
preds12_t <- readRDS(paste0("model_predicting_results/preds12_t_n", n, ".rds"))
preds23_t <- readRDS(paste0("model_predicting_results/preds23_t_n", n, ".rds"))

# Combine with predicted values
preds_t_comb <- preds12_t |> 
    left_join(true_values12, by = "t") |>
    mutate(pair = "z1 vs z2") |> 
    bind_rows(
      preds23_t |> 
        left_join(true_values23, by = "t") |>
        mutate(pair = "z2 vs z3")
    ) |> 
    mutate(
      win_low = logit_ci(win_prob, win_prob_se)[[1]],
      win_up = logit_ci(win_prob, win_prob_se)[[2]],
      loss_low = logit_ci(loss_prob, loss_prob_se)[[1]],
      loss_up = logit_ci(loss_prob, loss_prob_se)[[2]],
      pair = factor(pair, levels = c("z1 vs z2", "z2 vs z3"), labels = c(paste0("Z[1]", "~vs~", "Z[2]"), paste0("Z[2]", "~vs~", "Z[3]")))
    ) |>     
    group_by(
        pair, t
    ) |> 
    summarize(
        win_prob_true = mean(win_prob0),
        win_prob_est = mean(win_prob),
        win_bias = mean(win_prob - win_prob0),
        win_sd = sd(win_prob),
        win_se = mean(win_prob_se),
        win_coverage = mean(win_low <= win_prob0 & win_prob0 <= win_up, na.rm = TRUE),
        loss_prob_true = mean(loss_prob0), 
        loss_prob_est = mean(loss_prob),
        loss_bias = mean(loss_prob - loss_prob0),
        loss_sd = sd(loss_prob),
        loss_se = mean(loss_prob_se),
        loss_coverage = mean(loss_low <= loss_prob0 & loss_prob0 <= loss_up, na.rm = TRUE)
    ) |> 
    mutate(
        n = fct(paste0("n == ", n)),
        .before = 1
    ) 

preds_stats <- preds_stats |> bind_rows(preds_t_comb)

}
```

Plot win-loss probabilities

```
# Decide if you want to use this color scheme
color_scheme <- c("win" = "darkred", "loss" = "#0479A8")

# preds_stats |> count(pair)

preds_stats |> 
  mutate(
    pair = case_when(
      pair == "Z[1]~vs~Z[2]" ~ "Pair 1",
      pair == "Z[2]~vs~Z[3]" ~ "Pair 2"
    )
  ) |> 
  ggplot(aes(x = t)) +
  geom_ribbon(aes(ymin = win_prob_est - win_sd, ymax = win_prob_est + win_sd, fill = "1"), alpha = 0.2) +
  geom_ribbon(aes(ymin = loss_prob_est - loss_sd, ymax = loss_prob_est + loss_sd, fill = "2"), alpha = 0.2) +
  geom_line(aes(y = win_prob_est, color = "1"), linewidth = 1.2) +
  geom_line(aes(y = loss_prob_est, color = "2"), linewidth = 1.2) +
  geom_line(aes(y = win_prob_true), linetype = 3, linewidth = 1) +
  geom_line(aes(y = loss_prob_true), linetype = 3, linewidth = 1) +
  scale_x_continuous(limits = c(0, 1), breaks = seq(0, 1.5, by = 0.5)) +
  scale_color_discrete(labels = c("1" = "Win", "2" = "loss"), l = 60) +
  scale_fill_discrete(labels = c("1" = "Win", "2" = "loss"), l = 60) +
  labs(y = expression(w("t | z, z"^"*")), 
       color = NULL, fill = NULL) +
  # facet_grid(n ~ pair, labeller = label_parsed) +
  facet_grid(n ~ pair) +
  theme_minimal() +
  theme(
    legend.position = "top" 
  )
  
ggsave("figures/simu_win_loss_probs.png", width = 8, height = 9.5)
ggsave("figures/simu_win_loss_probs.eps", device = cairo_ps, width = 8, height = 9.5)
```

### Example with misspecified models

Misspecified model fitting and prediction

```
source("basic_functions.R")
source("model_parameter_specification.R")
# Parameter specification
lambdaH <- 0.5
lambdaD <- 0.2
betaD <- c(0.5, 0, -0.5)
betaH <- c(0.1, 0, -0.5)
# New covariates
z1 <- c(1, 0, 0)
z2 <- c(0, 0, 0)


n <- 1000
set.seed(12345)
# Generate data with mispecified model
df_miss <- generate_gb_data_misp(n, betaH, betaD, lambdaH, lambdaD, kappa, lambdaC, fmin, fmax)

# Fit PW models
mod_fit_miss <- pwreg1(df_miss$id, df_miss$time, df_miss$status, df_miss[, 4:ncol(df_miss)], eps = 1e-8)
# an earlier version for calculation of score residuals
mod_fit_miss0 <- pwreg(df_miss$id, df_miss$time, df_miss$status, as.matrix(df_miss[, 4:ncol(df_miss)]))

# Score residuals
score_resids <- score.proc(mod_fit_miss0)


# Fit Cox model on time to first event
# subset to first events
df_first <- df_miss |> 
  group_by(id) |> 
  slice_min(order_by = time, n = 1, with_ties = FALSE) |> 
  ungroup()

# df_first |> count(status)

cox_fit <- coxph(Surv(time, status > 0) ~ Z1 + Z2 + Z3, data = df_first)
# Calculate and plot schoenfeld residuals
score_resids_cox <- cox.zph(cox_fit)
score_resids_cox

# ?plot.cox.zph

# Plot
postscript("figures/two_panel_residuals.eps", width = 10, height = 5,
           horizontal = FALSE, onefile = FALSE, paper = "special")
par(mfrow = c(1, 2))
plot(score_resids_cox, var = 1, main = "Z1", ylab = "Schoenfeld Residuals")
plot(score_resids, 1, xlim = c(0, 3), ylim = c(-2, 2), ylab = "Cumulative win scores")
abline(h = 0, lty = 3)
dev.off()
# Use a simulation to calculate the true win probabilities over time
# comparing z1 against z2


# ?pwreg.score
preds <- predict(mod_fit_miss, z1, z2, contrast = TRUE)

# True values
win_loss_true <- readRDS("simulated_datasets/true_win_loss_prob_under_nonPW.rds")

preds |>
  ggplot(aes(x = time)) +
  geom_line(aes(y = win, color = "Win"), size = 1) +
  geom_line(data = win_loss_true,
            aes(y = win_prob, x = time, color = "Win"), size = 1, linetype = "dashed") +
  geom_line(aes(y = loss, color = "Loss"), size = 1) +
  geom_line(data = win_loss_true,
            aes(y = loss_prob, x = time, color = "Loss"), size = 1, linetype = "dashed") +
  scale_x_continuous("t",
    limits = c(0, 3)
  ) +
    # scale_color_discrete(breaks = c("Win", "Loss")) +
  labs(y = expression(w("t | z, z"^"*")),
              color = NULL, fill = NULL) +
  # facet_grid(n ~ pair, labeller = label_parsed) +
  theme_minimal() +
  theme(
    legend.position = "top"
  )

ggsave("figures/misspecified_model_win_loss_probs.png", width = 8, height = 4)
```

### Table 1: Inference of \(w(t\mid z, z^\*)\) at specific \(t\)’s

Collect results on win probabilities

```
# Time points to make inference on
ts <- c(0.05, 0.1, 1, 4)
# Take the results for these time points
all_stats <- preds_stats |> 
  filter(
    t %in% ts
  ) |> 
  mutate(
    n = parse_number(as.character(n)),
    # remove label of pair
    pair = factor(pair, labels = c("z1 vs z2", "z2 vs z3"))
  )
# Subset to the win statistics
win_stats <- all_stats |> 
  select(n, pair, t, win_prob_true, win_prob_est, win_bias, win_sd, win_se,  win_coverage) |> 
  pivot_wider(
    id_cols = c(n, t),
    names_from = pair,
    values_from = c(win_prob_true,  win_bias, win_sd, win_se, win_coverage)
  ) |> 
  select(
    n, t,
    contains("z1 vs z2"),
    contains("z2 vs z3")
  ) |> 
  group_by(n) |> 
  mutate(
    n = if_else(row_number() == 1, as.character(n), "")
  ) |> ungroup() |> 
  mutate(
    across(3:12, ~ round(., 3))
  )

colnames(win_stats)[3:12] <- c("True1", "Bias1", "SE1", "SEE1", "Coverage1", "True2", "Bias2", "SE2", "SEE2", "Coverage2") 

# Output in latex code
latex_code <- kable(win_stats, format = "latex", booktabs = TRUE)

# Print LaTeX code
cat(latex_code)

kable(win_stats)
```

### Table S2: Inference of \(w(t\mid z^\*, z)\) at specific \(t\)’s

Collect results on loss probabilities

```
# Do the same for loss probabilities
loss_stats <- all_stats |> 
  select(n, pair, t, loss_prob_true, loss_prob_est, loss_bias, loss_sd, loss_se,  loss_coverage) |> 
  pivot_wider(
    id_cols = c(n, t),
    names_from = pair,
    values_from = c(loss_prob_true,  loss_bias, loss_sd, loss_se, loss_coverage)
  ) |> 
  select(
    n, t,
    contains("z1 vs z2"),
    contains("z2 vs z3")
  ) |> 
  group_by(n) |> 
  mutate(
    n = if_else(row_number() == 1, as.character(n), "")
  ) |> ungroup() |> 
  mutate(
    across(3:12, ~ round(., 3))
  )

colnames(loss_stats)[3:12] <- c("True1", "Bias1", "SE1", "SEE1", "Coverage1", "True2", "Bias2", "SE2", "SEE2", "Coverage2")

# Output in latex code
latex_code <- kable(loss_stats,  format = "latex", booktabs = TRUE)

# Print LaTeX code
cat(latex_code)

kable(loss_stats)
```

### Table 2: Inference on win odds and net benefits

Collect results on win odds and net benefits

```
source("basic_functions.R")
source("model_parameter_specification.R")

z1 <- c(1, 0, 0)
z2 <- c(0, 0, 0)
z3 <- c(-1, 0, 0)


# Calculate the true values ------------------------------
lambda <- (lambdaH^kappa + lambdaD^kappa)^{1/kappa}
# Cut-off time points (ensure a smooth curve)
times <- c(seq(0.05, 1, by = 0.05), seq(1.1, 2, by = 0.1), 2.2, 2.5, 3, 3.5, 4)
# True parameters for the Cox TFE model
gamma0 <- - beta0
Lambda0 <- lambda * times
# Survival functions for the TFE
Sz1t <- exp(- exp(sum(gamma0 * z1)) * Lambda0)
Sz2t <- exp(- exp(sum(gamma0 * z2)) * Lambda0)
Sz3t <- exp(- exp(sum(gamma0 * z3)) * Lambda0)
# Mu functions from PW model
mu12 <- exp(sum(beta0 * z1)) / (exp(sum(beta0 * z1)) + exp(sum(beta0 * z2)))
mu23 <- exp(sum(beta0 * z2)) / (exp(sum(beta0 * z2)) + exp(sum(beta0 * z3))) 
# Comparability (win or loss) probabilities
comp_probs12 <- 1 - Sz1t * Sz2t
comp_probs23 <- 1 - Sz2t * Sz3t
# Win and loss probabilities
win_prob012 <- comp_probs12 * mu12
loss_prob012 <- comp_probs12 - win_prob012
tie_prob012 <- 1 - win_prob012 - loss_prob012
win_prob023 <- comp_probs23 * mu23
loss_prob023 <- comp_probs23 - win_prob023
tie_prob023 <- 1 - win_prob023 - loss_prob023

# Calculate net benefits
nb012 <- win_prob012 - loss_prob012
nb023 <- win_prob023 - loss_prob023
# Calculate win odds
wo012 <- (win_prob012 + 0.5 * tie_prob012) / (loss_prob012 + 0.5 * tie_prob012)
wo023 <- (win_prob023 + 0.5 * tie_prob023) / (loss_prob023 + 0.5 * tie_prob023)
# Tibble data frames for true values to be merged with simulated ones
true_values12 <- tibble(t = times, nb0 = nb012, wo0 = wo012)
true_values23 <- tibble(t = times, nb0 = nb023, wo0 = wo023)


# Read in the prediction results
preds_stats <- tibble(NULL)
for (n in c(100, 200, 500, 1000)){
# for (n in c(100)){
preds12_t <- readRDS(paste0("model_predicting_results/preds12_t_n", n, ".rds"))
preds23_t <- readRDS(paste0("model_predicting_results/preds23_t_n", n, ".rds"))


# Combine with predicted values

preds_t_comb <- preds12_t |> 
    left_join(true_values12, by = "t") |>
    mutate(pair = "z1 vs z2") |> 
    bind_rows(
      preds23_t |> 
        left_join(true_values23, by = "t") |>
        mutate(pair = "z2 vs z3")
    ) |> 
    mutate(
      pair = factor(pair, levels = c("z1 vs z2", "z2 vs z3"), labels = c(paste0("Z[1]", "~vs~", "Z[2]"), paste0("Z[2]", "~vs~", "Z[3]")))
    ) |> # CIs correct
    group_by(
        pair, t
    ) |> 
    summarize(
        nb0 = mean(nb0),
        nb_est = mean(nb),
        nb_bias = mean(nb - nb0),
        nb_sd = sd(nb),
        nb_coverage = mean(nb_low <= nb0 & nb0 <= nb_high),

        wo0 = mean(wo0),
        wo_est = mean(wo),
        wo_bias = mean(wo - wo0),
        wo_sd = sd(wo),
        wo_coverage = mean(wo_low <= wo0 & wo0 <= wo_high)
    ) |> 
    mutate(
        n = fct(paste0("n == ", n)),
        .before = 1
    ) 

preds_stats <- preds_stats |> bind_rows(preds_t_comb)

}
# preds_stats |> View()

ts <- c(0.1, 0.5, 1, 4)


all_stats <- preds_stats |> 
  filter(
    t %in% ts
  ) |> 
  mutate(
    n = parse_number(as.character(n)),
    # remove label of pair
    pair = factor(pair, labels = c("z1 vs z2", "z2 vs z3"))
  )


contrast_stats <- all_stats |> 
  select(n, pair, t, nb0, nb_bias, wo0, wo_bias, coverage = wo_coverage) |> 
  pivot_wider(
    id_cols = c(n, t),
    names_from = pair,
    values_from = c(nb0, nb_bias, wo0, wo_bias, coverage)
  ) |> 
  select(
    n, t,
    contains("z1 vs z2"),
    contains("z2 vs z3")
  ) |> 
  group_by(n) |> 
  mutate(
    n = if_else(row_number() == 1, as.character(n), "")
  ) |> ungroup() |> 
  mutate(
    across(3:12, ~ round(., 3))
  )

colnames(contrast_stats)[3:12] <- c("True_NB1", "Bias_NB1", "True_WO1", "Bias_WO1", "CP1", 
                                "True_NB2", "Bias_NB2", "True_WO2", "Bias_WO2", "CP2") 

# Output in latex code
latex_code <- kable(contrast_stats, format = "latex", booktabs = TRUE)

# Print LaTeX code
cat(latex_code)


kable(contrast_stats)
```

### Reproducible code example

MRE included in paper

```
devtools::install_github("lmaowisc/WR") # Install WR from GitHub
library(WR)
library(tidyverse) # For data wrangling and visualization
# Generate data in long format
n <- 10
df_y <- tibble(
  id = c(1, 1, 2, 3, 3, 4, 5, 5, 6, 7, 8, 9, 9, 10),
  time = c(0.2, 2, 2.3, 1, 4.5, 3.6, 2.2, 3.2, 5, 2.1, 3.8, 1.2, 4.5, 2.3),
  # status = 1 for death, 2 for hospitalization, 0 for censoring
  status = c(2, 1, 0, 2, 0, 1, 2, 1, 1, 0, 1, 2, 1, 0)
)
# Covariates
set.seed(123)
Z <- tibble(
  id = 1:n,
  Z1 = rnorm(n),
  Z2 = rbinom(n, 1, 0.5)
)
# Merge by id
df <- left_join(df_y, Z, by = "id")

# Fit the model
obj <- pwreg1(df$id, df$time, df$status, df[, 4:ncol(df)], eps = 1e-8)
# Calculate predictions
# Specify covariate vectors for comparison
z1 <- c(1, 0)
z2 <- c(0, 0)
# Predict win-loss probabilities, along with 
# win ratio, win odds, and net benefit
preds <- predict(obj, z1 = z1, z2 = z2, contrast = TRUE)
preds[1:9] # Basic output
#> # A tibble: 9 × 9
#>    time   win win_se win_high win_low   loss loss_se loss_high loss_low
#>   <dbl> <dbl>  <dbl>    <dbl>   <dbl>  <dbl>   <dbl>     <dbl>    <dbl>
#> 1   0.2 0.117  0.123    0.578  0.0126 0.0616  0.0652     0.374  0.00716
#> 2   1   0.223  0.172    0.668  0.0393 0.118   0.116      0.542  0.0148 
#> 3   1.2 0.315  0.244    0.808  0.0478 0.166   0.161      0.659  0.0202 
#> 4   2.1 0.315  0.244    0.808  0.0478 0.166   0.161      0.659  0.0202 
#> 5   2.2 0.409  0.253    0.843  0.0819 0.216   0.179      0.686  0.0336 
#> 6   2.3 0.409  0.253    0.843  0.0819 0.216   0.179      0.686  0.0336 
#> 7   3.6 0.529  0.232    0.875  0.154  0.280   0.187      0.705  0.0591 
#> 8   3.8 0.609  0.140    0.831  0.329  0.321   0.167      0.679  0.0958 
#> 9   5   0.652  0.115    0.834  0.410  0.344   0.119      0.596  0.157  
preds[c(1, 10:19)] # Additional output by contrast = TRUE
#> # A tibble: 9 × 9
#>    time    wr wr_low wr_high     nb nb_low nb_high    wo wo_low wo_high z_nb_wo
#>   <dbl> <dbl>  <dbl>   <dbl>  <dbl>  <dbl>   <dbl> <dbl>  <dbl>   <dbl>   <dbl>
#> 1   0.2  1.89  0.685    5.24 0.0551 -0.136   0.242  1.12  0.761    1.64   0.564
#> 2   1    1.89  0.685    5.24 0.105  -0.168   0.364  1.24  0.712    2.14   0.752
#> 3   1.2  1.89  0.685    5.24 0.149  -0.241   0.497  1.35  0.612    2.98   0.742
#> 4   2.1  1.89  0.685    5.24 0.149  -0.241   0.497  1.35  0.612    2.98   0.742
#> 5   2.2  1.89  0.685    5.24 0.193  -0.239   0.561  1.48  0.615    3.56   0.873
#> 6   2.3  1.89  0.685    5.24 0.193  -0.239   0.561  1.48  0.615    3.56   0.873
#> 7   3.6  1.89  0.685    5.24 0.250  -0.211   0.620  1.67  0.652    4.26   1.07 
#> 8   3.8  1.89  0.685    5.24 0.287  -0.132   0.619  1.81  0.767    4.25   1.35 
#> 9   5    1.89  0.685    5.24 0.308  -0.145   0.654  1.89  0.746    4.78   1.34
```

## Real example

### Table 3: Summary of the HF-ACTION dataset

Load and summarize the HF-ACTION dataset

```
library(WR)
library(survival)
library(tidyverse) # For data wrangling and visualization
library(gtsummary)
library(labelled)
library(knitr)

# Load the data
# Dataset non-public
# Can file a request to NHLBI BioLINCC: https://biolincc.nhlbi.nih.gov/studies/hf-action/
df0 <- read_csv("HF-ACTION/hf_action_base.csv") |> 
  select(patid, time, status, trt_ab, age, sex, racec, etiology, bmi, cpxdur, nyhacl, sixmwlkd, bestlvef, 
         creatnin) |> 
  arrange(patid, time, status) |> 
  group_by(patid, status) |> 
  slice(1) |> 
  arrange(patid, time) |> 
  ungroup()

# 1 age Num 8 Age at randomization.
# 2 bmi Num 8 Body Mass Index.
# 3 creatnin Num 8 Creatinine in mg/dL.
# 4 sex Num 8 1=Men; 2=Women
# 5 basehr Num 8 Heart rate at baseline (bpm).
# 6 sbp Num 8 Systolic blood pressure (mmHg).
# 7 dbp Num 8 Diastolic blood pressure (mmHg).
# 8 nyhacl Num 8 Baseline NHYA class.
# 39 racec Num 8 1=Black, 2=White, 3=Asian, Amer Ind, Pac. Isl
# 50 bestlvef Num 8 Best available baseline LVEF
# 15 sixmwlkd Num 8 Six-minute walk distance (meters), subjects able to walk.
# 16 sixmwlks Num 8 Six minute walk: symptomatic?
# 17 vevco2 Num 8 VeVCO2 slope, CPX test.
# 18 cpxdur Num 8 Exercise duration, CPX test (minutes).
# 92 etiology Num 8 Ischemic or non-ischemic etiology.

df <- df0 |> 
  mutate(
    sex = factor(sex, levels = c(1, 2), labels = c("Male", "Female")),
    racec = factor(racec, levels = c(1, 2, 3, 4), labels = c("Non-White", "White", "Non-White", "Non-White")),
    trt_ab = factor(trt_ab, levels = c(0, 1), labels = c("UC", "Training")),
    nyhacl = factor(nyhacl, levels = c(2, 3, 4), labels = c("II", "III", "IV")),
    etiology = factor(etiology, levels = c(1, 2), labels = c("Ischemic", "Non-ischemic"))
  )

# Label variables
var_label(df) <- list(
  patid = "Patient ID",
  time = "Time",
  status = "Status",
  trt_ab = "Training vs UC",
  age = "Age (years)",
  bmi = "Body Mass Index",
  creatnin = "Creatinine (mg/dL)",
  sex = "Sex",
  # basehr = "Heart rate (bpm)",
  # sbp = "Systolic blood pressure (mmHg)",
  # dbp = "Diastolic blood pressure (mmHg)",
  nyhacl = "NYHA class",
  racec = "Race",
  bestlvef = "LVEF (%)",
  sixmwlkd = "6MWD (meters)",
  # sixmwlks = "Six-minute walk: symptomatic?",
  # vevco2 = "VeVCO2 slope",
  cpxdur = "CPX duration (min)",
  etiology = "Etiology"
)

# maximum follow-up
range(df$time[df$status != 2]) / 365.25 # 6.863 years

min(df$time) / 365.25 # 6.863 years

# Summary table
tbl_hfone <- df |> 
  group_by(patid) |> 
  summarize(
    `Death (Outcome)` = any(status == 1),
    `Hospitalization (Outcome)` = any(status == 2)
  ) |> 
  left_join(df |> select(-c(time, status)) |> group_by(patid) |> slice(1), by = "patid") |>
  tbl_summary(
    by  = trt_ab,
    include = c(age, sex, racec, etiology,  bmi, nyhacl, cpxdur, sixmwlkd, bestlvef, `Death (Outcome)`, `Hospitalization (Outcome)`)
    ) |> 
  add_overall()


# Convert the gtsummary table to a gt table
gt_table <- as_gt(tbl_hfone)

# Output the LaTeX code for the table
latex_code <- gt::as_latex(gt_table)

# Print the LaTeX code to the console
cat(as.character(latex_code))
```

### Inital PW model

Fit the initial PW model

```
# Fit PW model
df1 <- df |> 
  mutate(
    time = time / 365.25,
    sixmwlkd = sixmwlkd / 100, # normalized to 100m
    train = (trt_ab == "Training"),
    female = (sex == "Female"),
    bmi = pmin(bmi, 60),
    nonwhite = (racec != "White"),
    ischmeic = (etiology == "Ischemic"),
    nyhacl3 = (nyhacl == "III"),
    nyhacl4 = (nyhacl == "IV")
  ) |> 
  select(
    patid, time, status, train, age, female, nonwhite, ischmeic, bmi, cpxdur, nyhacl3, nyhacl4, sixmwlkd, bestlvef
  )

# Fit PW model
obj <- pwreg1(df1$patid, df1$time, df1$status, df1[, 4:ncol(df1)])

# Extract coefficients and standard errors
beta <- obj$beta
se <- sqrt(diag(obj$Var))


# Calculate residuals
resids <- residuals(obj)

# Residual analysis
resids_MZ <- resids |> 
  select(id, M) |> 
  left_join(obj$Zn) |> 
  pivot_longer(
    c(cpxdur, sixmwlkd, bmi, bestlvef), 
    names_to = "var", 
    values_to = "value") |> 
  mutate(
    var = factor(
      var, levels = c("cpxdur", "sixmwlkd", "bmi", "bestlvef"),
      labels = c("CPX duration (min)", "6MWD (100m)", "BMI", "LVEF (%)")
      )
    )

# Plot win residuals
pw_resids <- resids_MZ |> 
  # filter(var != "nyhacl") |>
  ggplot(aes(x = value, y = M)) +
  geom_point(alpha = 0.2) +
  geom_smooth(method = "gam", formula = y ~ s(x, k = 200), se = FALSE) +
  facet_wrap(~ var, scales = "free") +
  labs(
    x = NULL,
     y = "Win residuals"
  ) +
  theme_minimal()

pw_resids

# ggsave("figures/pw_resids.png", width = 8, height = 6)
# ggsave("figures/pw_resids.eps", device = cairo_ps, width = 8, height = 6)


#### Cox model residuals

# Fit Cox model for TFE
df_tfe1 <- df1 |> 
  group_by(patid) |>
  arrange(time) |> 
  slice(1)

cox_obj <- coxph(Surv(time, status > 0) ~ train + age + female + nonwhite + ischmeic +  bmi + nyhacl3 + nyhacl4 + cpxdur + sixmwlkd + bestlvef, data = df_tfe1)

# nonmissing covariates
cox_Z <- model.matrix(cox_obj)

# Residual analysis for Cox model (Figure S1)
resids_MZ_cox <- 
  tibble(M = residuals(cox_obj, type = "martingale") ,
         cpxdur = cox_Z[, "cpxdur"],
         sixmwlkd = cox_Z[, "sixmwlkd"],
         bmi = cox_Z[, "bmi"],
         bestlvef = cox_Z[, "bestlvef"]
         )  |> 
  pivot_longer(
    c(cpxdur, sixmwlkd, bmi, bestlvef), 
    names_to = "var", 
    values_to = "value") |> 
  mutate(
    var = factor(
      var, levels = c("cpxdur", "sixmwlkd", "bmi", "bestlvef"),
      labels = c("CPX duration (min)", "Six-minute walk distance (m)", "BMI", "Best LVEF (%)")
      )
    )

# Plot residual vs covariates
cox_resids <- resids_MZ_cox |> 
  # filter(var != "nyhacl") |>
  ggplot(aes(x = value, y = M)) +
  geom_point(alpha = 0.2) +
  geom_smooth(method = "gam", formula = y ~ s(x, k = 200), se = FALSE) +
  facet_wrap(~ var, scales = "free") +
  labs(
    x = NULL,
     y = "Martingale residuals (TFE)"
  ) +
  theme_minimal()


cox_resids

# ggsave("figures/cox_resids.png", width = 8, height = 6)
# ggsave("figures/cox_resids.eps", device = cairo_ps, width = 8, height = 6)
#
```

### Figure 2 and Table 4

Refit model with cpxdur thresholded at 20 mins

```
# Refit model with cpxdur thresholded at 20 mins
df2 <- df1 |> 
  mutate(
    cpxdur = pmin(cpxdur, 20)
  )

obj2 <- pwreg1(df2$patid, df2$time, df2$status, df2[, 4:ncol(df2)])

# Re-do residual analysis
resids2 <- residuals(obj2)

resids_MZ2 <- resids2 |> 
  select(id, M) |> 
  left_join(obj2$Zn) |> 
  pivot_longer(
    c(cpxdur, sixmwlkd, bmi, bestlvef), 
    names_to = "var", 
    values_to = "value") |> 
  mutate(
    var = factor(
      var, levels = c("cpxdur", "sixmwlkd", "bmi", "bestlvef"),
      labels = c("CPX duration (min)", "6MWD (100m)", "BMI", "LVEF (%)")
      )
    )

# Residual plot
pw_resids2 <- resids_MZ2 |> 
  # filter(var != "nyhacl") |>
  ggplot(aes(x = value, y = M)) +
  geom_point(alpha = 0.2) +
  geom_smooth(method = "gam", formula = y ~ s(x, k = 200), se = FALSE) +
  facet_wrap(~ var, scales = "free") +
  labs(
    x = NULL,
     y = "Win residuals"
  ) +
  theme_minimal()

# # pw_resids2
# 
# ggsave("figures/pw_resids2.png", width = 8, height = 6)
# ggsave("figures/pw_resids2.eps", device = cairo_ps, width = 8, height = 6)

library(patchwork)
# Combine residual plots from initial and final models
pw_resids <- pw_resids + ggtitle("Initial model") + 
  theme(plot.title = element_text(face = "bold"))
pw_resids2 <- pw_resids2 + ggtitle("Final model") +
  theme(plot.title = element_text(face = "bold"))

pw_resids / pw_resids2

# Save plots
# ggsave("figures/pw_resids_all.png", width = 8, height = 9.8)
# ggsave("figures/pw_resids_all.eps", device = cairo_ps, width = 8, height = 9.8)

# Tabulate regression coefficients

beta <- obj2$beta
se <- sqrt(diag(obj2$Var))

reg_tbl <- tibble(
 term = c("Training vs UC", "Age (years)",  "Female vs Male", "Non-White vs White", "Ischemic vs Non-ischemic", "BMI", "CPX duration (min)", "NYHA class III vs II", "NYHA class IV vs II", "Six-minute walk distance (m)", "Best LVEF (%)"),
 WR = round(exp(beta), 2),
 `95% CI` = paste0("[", round(exp(beta - 1.96 * se), 2), ", ", round(exp(beta + 1.96 * se), 2), "]"),
 p_value = scales::pvalue(2 * (1 - pnorm(abs(beta / se))))
)


kable(reg_tbl, format = "latex", booktabs = TRUE)
```

### Table S3: Cox model results

Cox model results

```
# Get time to first event
df_tfe2 <- df2 |> 
  group_by(patid) |>
  arrange(time) |> 
  slice(1)

cox_obj2 <- coxph(Surv(time, status > 0) ~ train + age + female + nonwhite + ischmeic +  bmi + nyhacl3 + nyhacl4 + cpxdur + sixmwlkd + bestlvef, data = df_tfe2)

# Tabulate Cox model results
cox_summary <- summary(cox_obj2)
cox_tbl <- tibble(
 term = c("Training vs UC", "Age (years)",  "Female vs Male", "Non-White vs White", "Ischemic vs Non-ischemic", "BMI", "CPX duration (min)", "NYHA class III vs II", "NYHA class IV vs II", "Six-minute walk distance (m)", "Best LVEF (%)"),
 HR = round(cox_summary$coefficients[, "exp(coef)"], 2),
 `95% CI` = paste0("[", round(cox_summary$conf.int[, "lower .95"], 2), ", ", round(cox_summary$conf.int[, "upper .95"], 2), "]"),
 p_value = scales::pvalue(cox_summary$coefficients[, "Pr(>|z|)"])
)
kable(cox_tbl, format = "latex", booktabs = TRUE)
```

### Figure 3: Predicted win-loss probabilities, win ratio, win odds, and net benefit

Predictions based on final model

```
# Plot the predicted win-loss probabilities, win ratio, win odds, and net benefit
# Take median of quantitative covariate except cpx dur
medians <- df2 |> 
  select(patid, age, bmi, sixmwlkd, bestlvef, cpxdur) |>
  group_by(patid) |>
  slice(1) |> 
  ungroup() |> 
  summarize(
    age = median(age, na.rm = TRUE),
    bmi = median(bmi, na.rm = TRUE),
    sixmwlkd = median(sixmwlkd, na.rm = TRUE),
    bestlvef = median(bestlvef, na.rm = TRUE),
    cpxdur_min = min(cpxdur, na.rm = TRUE)
  )

# a non-white, non-ischemic female patient of median age (59) under UC with median
# BMI (30), median six-minute walk distance (372m), and median best LVEF (25%)
z0 <- c(train = FALSE, age = medians$age, female = TRUE, nonwhite = TRUE, ischmeic = FALSE, bmi = medians$bmi, cpxdur = 1, nyhacl3 = TRUE, nyhacl4 = FALSE, sixmwlkd = medians$sixmwlkd, bestlvef = medians$bestlvef)

# Set up the covariate vectors
z1 <- z0
z1["cpxdur"] <- 5
z2 <- z0
z2["cpxdur"] <- 10
z3 <- z0
z3["cpxdur"] <- 15
z4 <- z0
z4["cpxdur"] <- 20

# Predictions
preds10 <- predict(obj2, z1, z0, contrast = TRUE)
preds21 <- predict(obj2, z2, z1, contrast = TRUE)
preds32 <- predict(obj2, z3, z2, contrast = TRUE)
preds43 <- predict(obj2, z4, z3, contrast = TRUE)

# Stack results for plotting
preds <- preds10 |> mutate(pair = "CPX: 5 mins vs 1 min", .before = time) |>
  bind_rows(
    preds21 |> mutate(pair = "CPX: 10 mins vs 5 mins", .before = time)
  ) |>
  bind_rows(
    preds32 |> mutate(pair = "CPX: 15 mins vs 10 mins", .before = time)
  ) |>
  bind_rows(
    preds43 |> mutate(pair = "CPX: 20+ mins vs 15 mins", .before = time)
  ) |>
  mutate(pair = factor(pair, levels = c("CPX: 5 mins vs 1 min", "CPX: 10 mins vs 5 mins", "CPX: 15 mins vs 10 mins", "CPX: 20+ mins vs 15 mins"))) |> 
  filter(
    time <= 4
  )
  
# Clean up results for plotting
preds_wl <- preds |> 
  select(pair, time, win, loss) |> 
  mutate(
   tie = 1 - win - loss 
  ) |> 
  pivot_longer(
    c(win, loss, tie), 
    names_to = "win_loss", 
    values_to = "prob"
    ) |> 
  mutate(
    win_loss = factor(win_loss, levels = c("tie", "win", "loss"), labels = c("Tie", "Win", "Loss"))
  )

# Plot the win-loss-tie fractions (left column)
fig_hf_wl <- preds_wl |> 
  ggplot(aes(x = time, y = prob, fill = win_loss)) +
  geom_area(alpha = 0.9) +
  facet_wrap(~ pair, ncol = 1) +
  scale_fill_manual(limits = c("Win", "Loss", "Tie"), values = c("Win" = "#F8766D", "Loss" = "#00BFC4", "Tie" = "grey")) +
  labs(x = "Time (years)", y = "Probability", fill = NULL) +
  theme_minimal() +
  theme(
    legend.position = "top" 
  )


# Plot the win ratio, win odds, and net benefit (right column)
fig_hf_contr <- preds |> 
  ggplot(aes(x = time, y = wr)) +
  geom_ribbon(aes(ymin = wr_low, ymax = wr_high, fill = "WR"), alpha = 0.2) +
  geom_ribbon(aes(ymin = wo_low, ymax = wo_high, fill = "WO-NB"), alpha = 0.2) + 
  geom_line(aes(y = wr, color = "WR"), linewidth = 1) +
  geom_line(aes(y = wo, color = "WO-NB"), linewidth = 1) +
  facet_wrap(~ pair, ncol = 1) +
  scale_y_continuous("Win ratio/odds", limits = c(1, 1.75),
      sec.axis = sec_axis(
        "Net benefit",
        breaks = c(0,  0.1, 0.2, 0.3),
        transform = ~ (. - 1) / (. + 1)            # Transformation: rescale to original range
    )) +
  scale_fill_manual(limits = c("WR", "WO-NB"), values = c("WR" = "#d95f02", "WO-NB"= "#2a87de"), 
                    labels = c("WR" = "Win ratio", "WO-NB" = "Win odds (left); Net benefit (right)")) +
  scale_color_manual(limits = c("WR", "WO-NB"), values = c("WR" = "#d95f02", "WO-NB"= "#2a87de"), 
                    labels = c("WR" = "Win ratio", "WO-NB" = "Win odds (left); Net benefit (right)")) +
  labs(x = "Time (years)", fill = NULL, color = NULL) +
  theme_minimal() +
  theme(
   legend.position = "top" 
  )


# Split the two columns by 40% and 60%
fig_hf_wl + fig_hf_contr  + plot_layout(widths = c(0.4, 0.6))

# Save plots
ggsave("figures/preds_hf.png", width = 8, height = 9.5)
ggsave("figures/preds_hf.eps", device = cairo_ps, width = 8, height = 9.5)
```

### Table S4: Predictions based on final model

Tabulate numerical results from Figure 3

```
# Extract predictions for years 1, 2, 3, and 4
preds_tbl <- preds |> 
  group_by(pair) |> 
  filter(time <= 1) |> 
  slice_tail(n = 1) |> 
  bind_rows(
    preds |> 
      group_by(pair) |> 
      filter(time <= 2) |> 
      slice_tail(n = 1),
    preds |>
      group_by(pair) |> 
      filter(time <= 3) |> 
      slice_tail(n = 1),
    preds |>
      group_by(pair) |> 
      filter(time <= 4) |> 
      slice_tail(n = 1)
    
  )

# Tabulate  results
preds_num <- preds_tbl |> 
  mutate(
    time = round(time),
   wl = str_c(round(win, 2), "/", round(loss, 2)),
   wo = str_c(round(wo, 2), " [", round(wo_low, 2), ", ", round(wo_high, 2), "]"),
   nb = str_c(round(nb, 2), " [", round(nb_low, 2), ", ", round(nb_high, 2), "]")
  ) |> 
  select(time, pair, wl, wo, nb) |> 
  group_by(time) |>
  mutate(
    time = if_else(row_number() == 1, as.character(time), "")
  )

# Output in LaTeX code
latex_code <- kable(preds_num ,  format = "latex", booktabs = TRUE)

# Print LaTeX code
cat(latex_code)

kable(preds_num )
```
